# A reversible alkaline water electrolyser for load-flexible power–H_2_ interconversion enabled by bifunctional catalyst

**DOI:** 10.1093/nsr/nwaf325

**Published:** 2025-08-12

**Authors:** Xiaoyu Yan, Yang Zhao, Le Ke, Xiaoyu Wu, Kai Zhao, Lingjiao Li, Xiaojuan Cao, Xiaoyi Jiang, Ying Yang, Gadi Rothenberg, Ning Yan

**Affiliations:** Key Laboratory of Artificial Micro- and Nano-Structures of Ministry of Education, School of Physics and Technology, Wuhan University, Wuhan 430072, China; Shenzhen Research Institute of Wuhan University, Shenzhen 518057, China; Key Laboratory of Artificial Micro- and Nano-Structures of Ministry of Education, School of Physics and Technology, Wuhan University, Wuhan 430072, China; Key Laboratory of Artificial Micro- and Nano-Structures of Ministry of Education, School of Physics and Technology, Wuhan University, Wuhan 430072, China; Shenzhen Research Institute of Wuhan University, Shenzhen 518057, China; Key Laboratory of Artificial Micro- and Nano-Structures of Ministry of Education, School of Physics and Technology, Wuhan University, Wuhan 430072, China; Shenzhen Research Institute of Wuhan University, Shenzhen 518057, China; Key Laboratory of Artificial Micro- and Nano-Structures of Ministry of Education, School of Physics and Technology, Wuhan University, Wuhan 430072, China; Shenzhen Research Institute of Wuhan University, Shenzhen 518057, China; Key Laboratory of Artificial Micro- and Nano-Structures of Ministry of Education, School of Physics and Technology, Wuhan University, Wuhan 430072, China; Shenzhen Research Institute of Wuhan University, Shenzhen 518057, China; Key Laboratory of Artificial Micro- and Nano-Structures of Ministry of Education, School of Physics and Technology, Wuhan University, Wuhan 430072, China; Shenzhen Research Institute of Wuhan University, Shenzhen 518057, China; Key Laboratory of Artificial Micro- and Nano-Structures of Ministry of Education, School of Physics and Technology, Wuhan University, Wuhan 430072, China; Shenzhen Research Institute of Wuhan University, Shenzhen 518057, China; Hubei Key Laboratory of Theory and Application of Advanced Materials Mechanics, Wuhan University of Technology, Wuhan 430070, China; Van`t Hoff Institute for Molecular Sciences (HIMS), University of Amsterdam, Amsterdam 1098 XH, The Netherlands; Key Laboratory of Artificial Micro- and Nano-Structures of Ministry of Education, School of Physics and Technology, Wuhan University, Wuhan 430072, China; Shenzhen Research Institute of Wuhan University, Shenzhen 518057, China

**Keywords:** electrochemical energy conversion, electrocatalysis, fuel cells, battery, electrolyser

## Abstract

A safe, efficient and affordable electrochemical energy-storage system at the grid scale is necessary for the high-level integration of renewable energy. As an ideal energy carrier, hydrogen can be generated via water electrolysis and converted back into electricity via fuel cells. Although conventional alkaline water electrolysers are robust and relatively cost-effective, membrane-based fuel cells are often costly in terms of both capital and operational expenses. Herein, we developed a single-compartment, membrane-free alkaline water electrolyser, enabling reversible power-to-H_2_ conversion. The NiOOH redox mediator, sandwiched by two bifunctional electrodes (one for hydrogen oxidation and oxygen reduction reactions, and the other for hydrogen evolution and oxygen evolution reactions), circumvented the use of vulnerable ion-conducting membranes while preventing the formation of H_2_–O_2_ gas mixtures. Owing to the decoupled anodic and cathodic reactions, high-purity H_2_ (>99%) was produced at both high (>500 mA cm^−2^) and low (<50 mA cm^−2^) current densities, offering excellent load flexibility. Importantly, it effectively works reversibly as a fuel cell, with a peak power density of 0.23 W cm^−2^. In addition, it was also capable of working as a Ni–H_2_ battery for short-duration energy storage, delivering a peak power density of 1.4 W cm^−2^ and a round-trip efficiency of 83.6%. The electrolyser was easily switched between different operational modes while retaining excellent performance in a 350-h endurance test.

## Teaser

A new electrochemical device with decoupled electrode reactions enables functions of both battery and reversible fuel cells for load-flexible power-H_2_ interconversion.

## INTRODUCTION

The transition to renewable energy (RE) requires reliable and affordable large-scale energy-conversion and storage solutions [1–3]. Today, >95% of the storage capacity worldwide comes from pumped hydro, which is highly dependent on local geographic features, but might induce ecological problems [4–6]. In this context, emerging electrochemical approaches have recently attracted increasing attention [7–9]. Particularly, ‘power-to-hydrogen-to-power’ conversion comes into the spotlight. As an ideal energy carrier, hydrogen can be produced via water electrolysis without a carbon footprint [10–13]. The alkaline water electrolyser (AWE) is the only widely commercialized and mature technology that has relatively low capital expenditures and operating expenses compared with emerging alternatives based on polymer membrane electrode assembly. AWE uses electrocatalysts comprising Earth-abundant elements and is much more resistant to ionic impurities present in the water feedstock, thus circumventing the integration of a water purification system [14–17]. However, coupling AWE with intermittent and fluctuating RE is intrinsically challenging owing to its poor load flexibility. The operation at part load (low current densities) causes a severe H_2_–O_2_ gas mixture, demanding complete shutdown of the AWE in industrial practice. For typical AWEs in the commercial market, the load range is often set at 20%∼100% of the nominal load; thus, achieving high RE penetration is problematic [18–20].

Another challenge lies in the ‘hydrogen-to-power’ step. Both proton-exchange membrane fuel cells (PEMFCs) and solid-oxide fuel cells (SOFCs) have demonstrated potential for generating electricity efficiently [21–23]. However, the use of a large number of noble metal catalysts together with the questionable long-term stability results in no application in grid-scale energy storage [24–26]. Besides, compared with batteries, such systems based on hydrogen are more suitable in the scenario of long-duration energy storage (from days to seasons), which suffers from high operational costs and slow response times [27–29]. It is rather challenging to cost-effectively offer energy shifting across various timescales and power-load capacities to achieve a high-RE yet resilient power system.

In light of these challenges, we herein developed a single-compartment, membrane-free AWE that enables efficient power–H_2_ interconversion in different application scenarios. Because of the decoupled anodic and cathodic reactions, the electrolyser produced high-purity H_2_ (>99%) at both high (>500 mA cm^−2^) and low (<50 mA cm^−2^) current densities, offering excellent load flexibility. In the H_2_-to-power cycle, it effectively worked as a fuel cell, showing a peak power density of 0.23 W cm^−2^, or worked as a Ni–H_2_ battery for short-duration energy storage, delivering a peak power density of 1.4 W cm^−2^ and a round-trip efficiency of 83.6%. This work demonstrates the importance of reactor engineering and might pave the way for real-life applications of energy storage via green hydrogen.

## RESULTS AND DISCUSSION

### Device configuration and bifunctional materials

**Figure 1.**
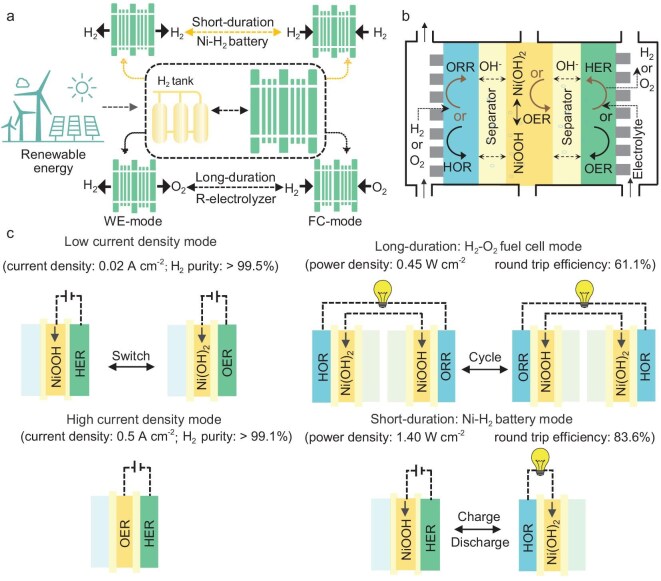
(a) Schematic of RE storage at different timescales using the proposed AWE, (b) the cell assembly of the device and (c) the working principles of the WE mode (low/high current density), fuel cell mode and Ni–H_2_ battery mode.

The developed electrolyser works in water electrolysis (WE), fuel cell or Ni–H_2_ battery mode for short-duration and long-duration energy storage, as depicted in Fig. 1a. It features a single compartment filled with aqueous KOH electrolyte (see Fig. 1b and Fig. S1). No ion-exchange membrane or membrane electrode assembly is used, thus resulting in a lower cost and greater robustness. The gas-diffusion electrode (GDE) is a bifunctional catalyst that is active for the hydrogen oxidation reaction (HOR) and oxygen reduction reaction (ORR), whereas the porous electrode (PE) contains a bifunctional catalyst for the hydrogen evolution (HER) and oxygen evolution reactions (OER). A Ni electrode, Ni(OH)_2_–NiOOH, is sandwiched between the GDE and PE in the middle to decouple the anodic and cathodic reactions or catalyse the OER at high loads. Thus, there are three basic reversible reactions, as shown below, and standard reduction potentials (*E*^0^) are given relative to the standard hydrogen electrode:


(1) \begin{eqnarray*}
\text{2}{{\text{H}}_{\text{2}}}\text{O } &+& \text{ 2}{{\text{e}}^{-}} \mathop {\mathop {\rightleftarrows} \limits_{{\mathrm{GDE}}} }\limits^{{\mathrm{PE}}}\, {{\text{H}}_{\text{2}}} + \text{ 2O}{{\text{H}}^{-}}\\ &&\quad\qquad ({{E}^{0}} = -0.83\, \text{ V});
\end{eqnarray*}

(2) \begin{eqnarray*}
{\mathrm{NiOOH}} &+& {{\mathrm{H}}_{\mathrm{2}}}{\mathrm{O}}+ {{\mathrm{e}}^ - } \mathop {{\rightleftarrows}} \limits {\mathrm{Ni}}{\left( {{\mathrm{OH}}} \right)_{\mathrm{2}}} + {\mathrm{O}}{{\mathrm{H}}^ - } \\
&&\qquad\qquad ({E^0} = \ + 0.49\, {\mathrm{ V}});
\end{eqnarray*}

(3) \begin{eqnarray*}
{\mathrm{2}}{{\mathrm{H}}_{\mathrm{2}}}{\mathrm{O}} + {{\mathrm{O}}_{\mathrm{2}}} + {\mathrm{4}}{{\mathrm{e}}^ - }\mathop {\mathop {{\rightleftarrows}} \limits_{{\mathrm{PE}}/{\mathrm{Ni}}} }\limits^{{\mathrm{GDE}}} {\mathrm{4O}}{{\mathrm{H}}^ - } \\
({E^0} = \ + 0.41\, {\mathrm{V}}).
\end{eqnarray*}

In WE mode, the device becomes a water electrolyser with the half reactions shown by Equations (1–3). At low current densities, the Ni electrode serves as the auxiliary electrode for temporally decoupling the HER and OER reactions. When the HER occurs at the PE, Ni(OH)_2_ on the Ni electrode oxidizes to form NiOOH, as shown by Equations (1) and (2) (see Fig. 1c). Conversely, during the OER at the PE, the oxidized NiOOH on the Ni electrode is reduced back to its initial state, as shown by Equations (2) and (3). At high current densities, the Ni electrode functions as an OER electrode, whereas the HER occurs on PE according to Equations (1) and (3).

In fuel cell mode, two single cells are connected in series to enable continuous operation and become the anode and cathode compartments. The Ni electrode, following the redox reaction shown by Equation (3), serves as the auxiliary electrode to physically decouple the anodic/cathodic reactions and circumvent the use of an ion-exchange membrane. The HOR and ORR proceed on GDEs during the discharging process, enabling the conversion of hydrogen into electricity in a similar way to a fuel cell (see Fig. 1c). Note that the operations feature a cyclic characteristic, as the potential bias between the two compartments must be swapped periodically when the auxiliary electrodes are electrochemically consumed. H_2_- and O_2_-rich electrolytes are circulated separately by pumps to avoid H_2_–O_2_ mixtures in the liquid phase. In the Ni–H_2_ battery mode, the device becomes a Ni–H_2_ battery with the charging/discharging half reactions shown by Equations (1) and (2). The GDE and PE work during the discharging and charging cycles, respectively. The detailed operation sequence is illustrated in Fig. 1c and further clarified in detail in Figs S2–S5.

**Figure 2.**
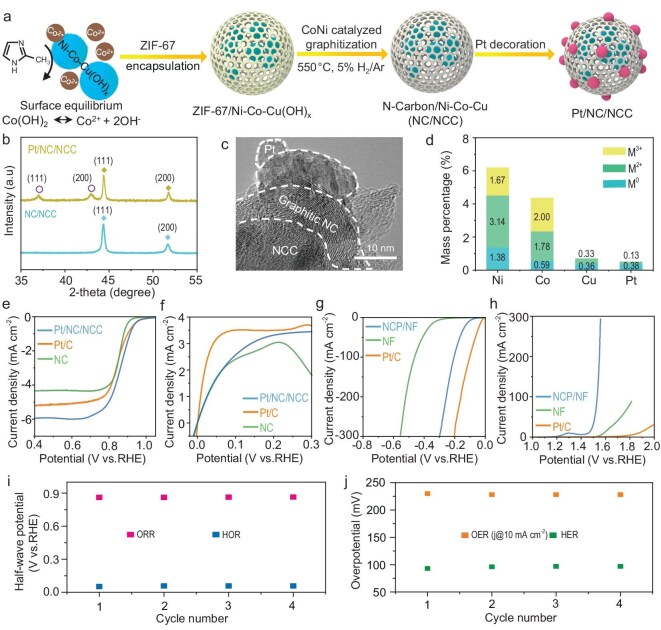
Synthetic procedure and characterization of the Pt/NC/NCC catalyst. (a) Representative illustration of the synthetic procedure, (b) XRD patterns, (c) TEM image, (d) relative content ratios of metals in different valence states, (e) ORR polarization curves, (f) HOR polarization curves, (g) HER polarization curves, (h) OER polarization curves, (i) half-wave potentials of the Pt/NC/NCC electrode cycling between the ORR and HOR and (j) overpotentials of the PE (nickel and cobalt phosphide (NCP)/nickel foam (NF)) cycles between the HER and OER.

The development of the HOR and ORR bifunctional catalysts is particularly challenging, as their high potential during the ORR often results in the (surface) oxidation of typical noble-metal-free HOR catalysts. To ensure robustness, we encapsulated a Ni–Co–Cu alloy, a good HOR catalyst with graphitic N-doped carbons (denoted as NC/NCC; see the schematic in Fig. 2a). This encapsulation was enabled by the coordination of 2-methylimidazole with dissolved cobalt cations (due to dissolution‒precipitation equilibrium) near the surface of Ni–Co–Cu(OH)*_x_*, forming a zeolitic imidazolate framework (ZIF-67) layer. Sequential pyrolysis led to the carbonization of ZIF-67 and the reduction of Ni–Co–Cu(OH)*_x_*, generating NC/NCC. We finally decorated the surface of the graphitic NC with 0.1 wt% Pt to further increase the bifunctional activity.

The X-ray diffraction (XRD) pattern of the NC/NCC sample is shown in Fig. 2b, in which the peaks at 44.3° and 51.7° are attributed to the (111) and (200) planes of the Co–Ni–Cu alloy [30–32]. After Pt decoration, new peaks at 37.1° and 43.0° appear, which belong to the Pt phase [33–35]. However, no diffraction peak of graphite is found, indicating that a small amount of NC formed on the surface of the NCC. To verify the existence of NC, we used Raman spectroscopy, as shown in Fig. S6. The two main peaks at 1345 and 1602 cm^−1^ are assigned to the D-band and G-band of carbon, respectively [36–38]. The morphology of Pt/NC/NCC was characterized via transmission electron microscopy (TEM), as shown in Fig. 2c and Fig. S7. The high-resolution TEM micrograph displays the hierarchical structure of three component phases: the lattice fringe of 0.214 nm corresponds to the (111) plane of the Ni–Co–Cu alloy, whereas that of 0.191 nm corresponds to the (200) facet of Pt [39,40]. The graphitic shells of NC on the surface of the NCC are also visible. The X-ray photoelectron spectroscopy data shown in Fig. S8 reveal the presence of Ni, Co, Cu, Pt, C and N, which confirms the formation of NCs with abundant pyridinic active sites for the ORR. The atomic ratios of Ni, Co, Cu and Pt were 6.20:4.37:0.69:0.51, as shown in Fig. 2d, further confirming the successful synthesis of Pt/NC/NCC. As the NC layer is <10 nm thick, all the Ni, Co and Cu present beneath it are detectable, which contributes to the HOR. Furthermore, the energy-dispersive X-ray spectroscopy elemental mappings shown in Fig. S9 reveal the formation of the NCC alloy.

This Pt/NC/NCC catalyst outperforms the commercial 20 wt% Pt on carbon (Pt/C) in the ORR, while its activity in the HOR is inferior to that of its Pt/C counterpart (see the voltammograms in Fig. 2e and f). The half-wave potential reached 0.863 V vs. RHE (reversible hydrogen electrode) in the ORR, with an electron-transfer number of 3.96 (see the Koutecky–Levich plot in Fig. S10) [41,42]. NC was a better support than conventional carbon, which was also active for the ORR and demonstrated a half-wave potential of 0.850 V vs. the RHE. For the HOR, the kinetic current density of Pt/NC/NCC was 4.95 mA cm^−2^ at an overpotential of 50 mV (see Fig. S11). The corresponding mass activity was 442.0 mA mg^−1^. NCC was also active for hydrogen oxidation, yet the breakdown potential was ∼0.21 V, suggesting that it alone was not suitable for HOR/ORR bifunctional applications, as the ORR cycle at high potential can cause surface oxidation of the alloy. The nickel and cobalt phosphide (NCP)/nickel foam (NF) PE delivered current densities of 10, 50 and 250 mA cm^−2^ in the HER at overpotentials of 93, 145 and 242 mV, respectively (see Fig. 2g). This performance approached that of commercial Pt/C and was much better than that of naked NF. In the OER, by contrast, NCP/NF drastically outperformed both Pt/C and NF (see Fig. 2h). To reach current densities of 10, 50 and 250 mA cm^−2^, NCP/NF required overpotentials of only 230, 280 and 323 mV, respectively. Both the Pt/NC/NCC and NCP/NF catalysts demonstrated excellent stability during frequent cycles among the two designated reactions. At a current density of 10 mA cm^−2^, the overpotentials of Pt/NC/NCC in the ORR and HOR did not change during the four cycles (see Fig. 2i and Fig. S12a and b). NCP/NF performed equally well in the OER and HER cycles (see Fig. 2j and Fig. S12c and d). These results suggest that both catalysts are not only bifunctionally active, but also suitable for cyclic operation between the two reactions.

To evaluate the antioxidation capability of Pt/NC/NCC, we carried out differential electrochemical mass spectrometry (DEMS). In the CV scan from 0.9 to 2.4 V vs. the RHE (Fig. S13) in a 0.1 M KOH electrolyte, three signals at m/z = 28, 44 and 32 were recorded for both Pt/C (dashed lines) and Pt/NC/NCC (solid lines). Although the oxidation products (CO and CO_2_) were observed in both samples, the onset potentials of both CO and CO_2_ evolution increased to 2.03 and 1.75 V vs. RHE, respectively, for Pt/NC/NCC, which were ∼730 and ∼540 mV higher than those of Pt/C. In fact, both Pt-catalysed carbon oxidation and electrochemical NC corrosion have been widely observed during the ORR. As our NC layer is highly graphitic, its resistance against oxidation at high anodic potentials is drastically improved, as reflected by the much higher corrosion potentials. The encapsulation of NCC by NC also increased the anticorrosion resistance of NCC, as demonstrated by the characterization results of the spent catalysts (*vide infra*). Pt/NC/NCC (2 wt%) was deposited on the catalyst layer of the GDE, as shown by the cross-sectional scanning electron microscopy (SEM) image in Fig. S14. PE is a piece of NF uniformly coated with NCP, as shown by the SEM image in Figs S15 and S16. The cross-sectional and surface images demonstrate that the NCP layer is composed of dense phosphide nanowires anchored on the surface of the NF.

### Three operation modes

**Figure 3.**
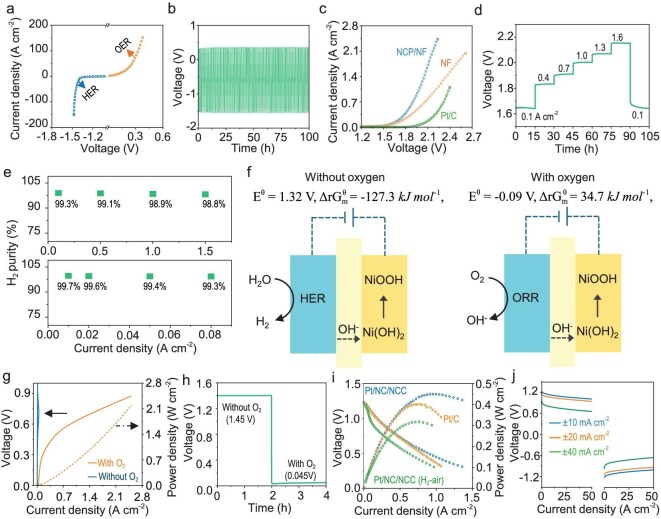
Electrochemical performance of the electrolyser in WE mode and fuel cell mode. (a) Polarization curves of the HER and OER at low current densities in WE mode, (b) cyclic stability curve at 50 mA cm^−2^ in WE, (c) polarization curve of the electrolyser at high current densities in WE mode and (d) multistep current stability curves of the electrolyser at high current densities. (e) H_2_ purity at different current densities. (f) Schematic of the oxygen compartment in fuel cell operation under different conditions, (g) polarization and electrolytic power density curves of the oxygen compartment with/without O_2_, (h) galvanostatic profile at 10 mA cm^−2^ with the injection of O_2_ at 2 h, (i) polarization and power density curves of the fuel cell in the discharging mode and (j) cyclic discharge curves of the fuel cell in the discharging mode. All tests were performed at 50°C in 30 wt% KOH.

In the electrochemical test of the electrolyser, we tested a short stack comprising two single cells to enable continuous operation in WE mode. At low current densities, the operation requires the HER to occur on the PE electrode, whereas the oxidation reaction of Ni(OH)_2_ occurs on the Ni electrode. In the polarization curve of a single water electrolyser using the NCP/NF PE (see Fig. 3a), the potential biases were 1.45, 1.47, 1.50 and 1.53 V at current densities of 10, 20, 50 and 100 mA cm^−2^, respectively. When the Ni(OH)_2_ conversion reached the set point, the current polarity was reversed so that the OER occurred on the PE electrode, whereas the reduction reaction of NiOOH occurred on the Ni electrode. The potential biases were 0.16, 0.22, 0.31 and 0.38 V at current densities of 10, 20, 50 and 100 mA cm^−2^, respectively. The stability test was performed at 50 mA cm^−2^, as shown in Fig. 3b, and no significant voltage increase was observed after 100 h. At high current densities, the operation requires the HER to occur on the PE electrode, whereas the OER occurs on the Ni electrode. The potential biases were 1.63, 1.83, 1.97 and 2.06 V at current densities of 0.1, 0.5, 1.0 and 1.5 A cm^−2^, respectively (see Fig. 3c). The stability test using the multistep current revealed that the potential stabilized rapidly after each current ramp, as shown in Fig. 3d. Because of the decoupled anodic and cathodic reactions, the electrolyser produced high-purity H_2_ (>99%) at both high current densities (>500 mA cm^−2^) and low current densities (<50 mA cm^−2^), as shown in Fig. 3e, and offered excellent load flexibility.

In fuel cell operation, two cells in the stack are connected in series. The hydrogen-purged cell still functions as a Ni–H_2_ battery (H_2_-cell), whereas the oxygen is purged into the other cell (O_2_-cell). In the O_2_-cell during the discharging cycle, Ni(OH)_2_ was oxidized into NiOOH, whereas the ORR occurred on the GDE. The Nernst potential of this compartment is +0.09 V, implying electrolytic operation. However, without O_2_, the cathodic reaction on the GDE would be the HER, and the applied electrolytic potential would be +1.32 V (see Fig. 3f and Fig. S17). The polarization curves shown in Fig. 3g and the galvanostatic profile of the O_2_-cell shown in Fig. 3h confirm that the ORR indeed improved the cell performance at a much lower potential.

Figure 3i shows the discharging performance of the in-series-connected stack. The open-circuit voltage is 1.23 V, which is identical to the Nernst potential of the fuel cell, confirming the ‘fuel-cell-like’ operation with decoupled ORR and HOR by the Ni(OH)_2_/NiOOH auxiliary electrode. The peak power density was 0.45 W cm^−2^ (nominally 0.23 W cm^−2^ for each cell), which was lower than that of the Ni–H_2_ battery and the theoretical result (see details in Fig. S18), as power consumption and parasitic loss occurred in the electrolytic reaction from the oxygen compartment. At constant current densities of ±10, ±20 and ±40 mA cm^−2^, the voltages were stabilized at 1.05, 0.99 and 0.81 V, respectively, with energy efficiencies of 70.8%, 66.8% and 54.6%, respectively (see Fig. 3j and Fig. S19). When the Ni(OH)_2_/NiOOH conversion reached the set point, the oxygen and hydrogen feed streams, together with the designated flowing electrolytes, were switched between the two cells, accompanied by a reversal of the current polarity. The longevity test shown in Fig. S20 shows a 120-h cycle operation at a constant current of ±10 mA cm^−2^. The negligible potential increase suggested the robustness of the fuel cell as well as the bifunctional stability of the Pt/NC/NCC electrode. We also evaluated the performance of the fuel cell by using air (CO_2_ was removed by using a CO_2_ scrubber; see Fig. S21). The peak power density decreased to 0.317 W cm^−2^ but was reasonable for real-life applications.

Figure S22a shows the single-cell performance in the Ni–H_2_ mode. The open-circuit voltage reached 1.32 V, which is identical to the theoretical Nernst potential. The peak power density reached 1.4 W cm^−2^, which is substantially higher than that of a commercial Ni–metal hydride battery (Ni–MH, 0.19 W cm^−2^). The galvanostatic charge/discharge test of the Pt/NCC/NC electrolyser at 10 mA cm^−2^ shown in Fig. S22b revealed distinct charge/discharge plateaus at ∼1.50/1.18 V, with a coulombic efficiency of 97.5% and a round-trip energy efficiency of 76.7%. The overpotential during charging increased when the charging current density increased and the coulombic efficiency decreased to 88.3% when the current density reached 40 mA cm^−2^. The excellent rate capacity was confirmed by measurements at various current densities (see Fig. S23). The calculated energy density was 208.4 Wh kg^−1^ (40 mA cm^−2^), 93.8% of which was retained after 10 000 cycles (see Fig. S24). These performance indicators confirmed the superior performance of the new configuration.

### Dynamic switching and efficiency analysis

**Figure 4.**
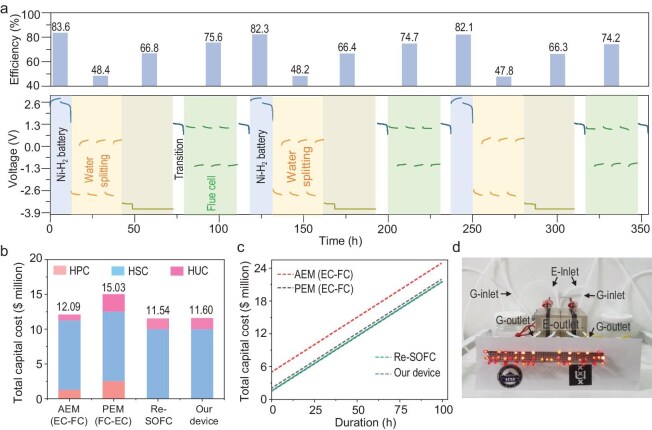
Dynamic switching of the AWE. (a) Continuous operation of the device at 10 mA cm^−2^ between the Ni–H_2_ battery, low-current-density water electrolysis (LCD-WE), high-current-density water electrolysis (HCD-WE) and fuel cell modes; (b) hydrogen production cost (HPC), hydrogen storage cost (HSC) and hydrogen utilization cost (HUC) comparison of various energy-storage devices; (c) total capital cost of various energy-storage devices from 1 and 100 h; (d) light-emitting diode (LED) bulbs powered by the short stack.

To simulate real-life applications in various energy-storage scenarios, we switched the short stack between different modes, as shown in Fig. 4a. The current density was set at 10 mA cm^−2^ in all the modes. The charge and discharge tests in the Ni–H_2_ battery delivered voltages of ∼2.8 and ∼2.4 V, respectively, as the device consisted of two batteries in series. During this short-duration energy-storage cycle, 151.2 and 126.4 J of electrical energy are stored and released, respectively. The nominal round-trip energy efficiency was 83.6%, which is comparable to that of typical battery systems.

When the AWE subsequently transitions to WE mode, it operates under either low-current-density or high-current-density conditions. The discharge in the previous Ni–H_2_ battery cycle resulted in the formation of Ni(OH)_2_ in both compartments. Upon switching to low-current-electrolysis mode, the HER is first initiated to convert Ni(OH)_2_ into NiOOH, followed by the OER. Throughout the cyclic electrolysis operation, the voltages for the HER and OER are −2.82 and +0.32 V, respectively. After 30 h of low-current-density electrolysis, 0.7 mmol of H_2_ is generated, which is equal to energy storage of 198.7 J. The nominal energy efficiency was 47.5%. In high-current-density electrolysis mode, Ni(OH)_2_ is initially oxidized to NiOOH, which subsequently acts as an active species that catalyses the OER. The combined voltage is 3.68 V. High-current-density electrolysis results in the formation of NiOOH in both compartments. Therefore, switching to fuel cell mode, with only one compartment initially working, converts NiOOH into Ni(OH)_2_. During the discharge cycle, the average operating voltage reached 1.12 V. After 30 h of discharging, 302.4 J of electrical energy was released. The nominal discharging energy efficiency was 75.6%.

For the technoeconomic analysis, the design of a large stack is shown in Fig. S25. The stack unit features two substacks connected in parallel, whereas the electrolysers in both substacks are connected in series. During WE, fuel cell or Ni–H_2_ operation modes, this configuration enables H_2_-related reactions (HOR or HER) in one substack, whereas the corresponding O_2_-related reactions (ORR or OER) proceed in the other substack, as shown in Figs S26–S28. To estimate the cost of the membrane-free AWE, we considered the hydrogen production cost, hydrogen storage cost (HSC) and hydrogen utilization cost (HUC) in the model of a 100-MW power station with a storage duration ranging from 1 to 100 h (see Fig. 4b and c). The costs of other typical energy-storage systems, including anion exchange membrane electrolysis cells-anion exchange membrane fuel cells (AEMEC-AEMFCs), proton exchange membrane electrolysis cells proton exchange membrane fuel cells (PEMEC-PEMFCs) and reversible SOFCs (Re-SOFCs), were also plotted as a reference. The membrane-free AWE is comparable to that of the Re-SOFC but outperforms other counterparts. The detailed calculations are shown in the SI. We also assembled an AWE with a relatively large electrode area (3 × 3 cm). Figure 4d and Fig. S29 show the lighting of 42 LEDs (power rating@1.43 W) in both fuel cell mode and Ni–H_2_ battery mode.

## CONCLUSION

In summary, a single-compartment, membrane-free AWE was developed, successfully achieving reversible power-to-H_2_ conversion. Owing to the decoupled anodic and cathodic reactions, high-purity H_2_ was produced at both high and low current densities, offering excellent load flexibility. Importantly, it effectively works reversibly as a fuel cell for long-duration energy storage and as a conventional Ni–H_2_ battery for short-duration energy storage. This electrolyser easily switches between two modes while retaining excellent performance. This work provides a new idea for designing a single device for energy storage covering various loads and timescales. This work also lays a preliminary foundation for the future application of the high-level integration of RE.

## Supplementary Material

nwaf325_Supplemental_File

## Data Availability

The data that support the plots of this paper and other findings of this study are available from the corresponding authors upon reasonable request.
