# Supplementary material for: A reversible alkaline water electrolyser for load-flexible power–H2 interconversion enabled by bifunctional catalyst
Source: Natl Sci Rev. 2025 Aug 12;12(10):nwaf325. doi: 10.1093/nsr/nwaf325 (PMC12462615; doi:10.1093/nsr/nwaf325)
Supplement: nwaf325_Supplemental_File [file nwaf325_supplemental_file.pdf]

Supplementary information for

A reversible alkaline water electrolyzer for load-flexible power-H_2 interconversion enabled by bifunctional catalyst

Xiaoyu Yan,[1,4] Yang Zhao,[1] Le Ke,[1,4] Xiaoyu Wu,[1,4] Kai Zhao,[1,4] Lingjiao Li,[1,4] Xiaojuan Cao,[1,4] Xiaoyi Jiang,[1,4] Ying Yang,[3] Gadi Rothenberg,[2] and Ning Yan [1,4*]

[1] Key Laboratory of Artificial Micro- and Nano-Structures of Ministry of Education, School of Physics and Technology, Wuhan University, Wuhan, 430072, China.

[2] Van`t Hoff Institute for Molecular Sciences (HIMS), University of Amsterdam, Amsterdam, 1098 XH, The Netherlands.

[3] Hubei Key Laboratory of Theory and Application of Advanced Materials Mechanics, Wuhan University of Technology, Wuhan, 430070, China.

[4] Shenzhen Research Institute of Wuhan University, Shenzhen, 518057, China.

*To whom the correspondence should be addressed: ning.yan@whu.edu.cn.

Supplementary information includes:

1. Materials and Device 3

1.1 Preparation of HOR/ORR bifunctional catalyst 3

1.2 Preparation of HER/OER bifunctional catalyst 4

1.3 $Ni(OH)_2$/NiOOH electrode 4

1.4 Rotating disk electrode (RDE) preparation..4

1.5 Gas diffusion electrode (GDE) and porous electrode (PE) preparation 4

1.6 electrolyzer assembly..5

2. Materials and electrode characterization 5

2.1 Materials characterizations 5

2.2 Electrocatalysts performance 5

2.3 Differential electrochemical mass spectrometry (DEMS) 6

3. electrolyzer test 6

3.1 Battery test 7

3.2 Fuel cell and electrolyzer test 7

4. Supplementary Figures 10

5. Cost evaluation...40

5.1 Electrolyzer cost...40

5.2 Energy storage cost comparison 40

6. Supplementary References...46

1. Materials and Device

$Co(NO_3)_2·6H_2O$, $Ni(NO_3)_2·6H_2O$, $Cu(NO_3)_2·3H_2O$, H_2PtCl_6, CH_4N_2O, NH_4F, 2-methylimidazole (2-MeIM) were supplied by Aladdin Chemical Reagent Ltd., China. All reagents were of analytical grade and used without further purification. Nafion™ 117 containing (~5%wt) solution was obtained from E.I. Du Pont Co., USA. KOH and absolute CH_3OH were purchased from Sinopharm Chemical Reagent, China. 20%wt Pt/C was obtained from Johnson Matthey Co., Ltd., USA. Nickel strips (thicknesses: 0.08 mm), Ni foam (NF, thicknesses: 1 mm) and carbon paper with gas diffusion layer (YLS-30T, thicknesses: 235 μm) were purchased from Sinera technology Co., Ltd, China. $Ni(OH)_2$ electrode (mass loading: 330 mg cm^{-2}) and polypropylene-based separator (YLD-GS, thicknesses: 0.15 ± 0.02 mm) were offered by Newcom Energy Materials, China. Commercial Ni-MH battery (AAA, 300 mAh) were supplied by Delipower Battery Co., Ltd., China. Deionized (DI) water was used in all materials preparations.

1.1 Preparation of HOR/ORR bifunctional catalyst

1.1.1 Preparation of NiCoCu(NCC)(OH)x

1.397 g $Ni(NO_3)_2$ $6H_2O$, 0.931 g $Co(NO_3)_2$ $6H_2O$, 0.097 g $Cu(NO_3)_2$ $3H_2O$, 1.2 g CH_4N_2O and 0.068g NH_4F were dissolved in 20 mL DI water and stirred for 10 min, and then the mixture was transferred into a 25 mL Teflon-lined stainless-steel autoclave. After 12 h of reaction at 120 ℃, the as-synthesized sample $NCC(OH)_x$ was washed several times with DI water and dried at 60 ℃ in vacuum for 6 h.

1.1.2 Preparation of 2D ZIF-67/NCC(OH)$_x$

1.64 g 2-MeIM was dissolved in a mixture of 10 mL deionized water and 15 mL methanol, and then 0.2 g $NCC(OH)_x$ was placed into above solution, which was stirred for 300 min at room temperature. The reacted sample with purple color was taken out and rinsed with methanol several times, and finally dried in an oven at 60 ℃ to obtain 2D ZIF-67/$NCC(OH)_x$ power.

1.1.3 Preparation of NC/NCC

The obtained powder was heated up and reduced at 550 ℃ for 2 h in a stream of 5% H_2/Ar.

1.1.4 Preparation of 2% Pt/NC/NCC

200 mg NC/NCC was added into 20 mL DI water. After vigorous stirring, 0.02 g mL^{-1} Na_2PtCl_6 (465 uL) solution was slowly added into the suspension. Under vigorous stirring at 65 ℃ for 12 h, all water was evaporated completely and the

residual balck powder was retrieved. The obtained powder was heated up and reduced at 200 ℃ for 2 h in a stream of 5% H_2/Ar.

1.2 Preparation of HER/OER bifunctional catalyst

Preparation of $NiCo(OH)_2$/NF. 0.582 g $Ni(NO_3)_2$ $6H_2O$, 0.582 g $Co(NO_3)_2$ $6H_2O$, 0.43 g CH_4N_2O, 0.032 g NH_4F were dissolved in 20 mL DI water and stirred for 10 min. The solution and a clean piece of NF were transferred into a 25 mL Teflon-lined stainless-steel autoclave. After 12 h of reaction at 120 ℃, the as-synthesized sample ($NiCo(OH)_2$/NF) was washed several times with DI water and dried at 60 ℃ in vacuum for 6 h.

Preparation of NiCoP/NF (NCP/NF). NaH_2PO_2 H_2O and $NiCo(OH)_2$/NF were respectively placed in two small porcelain boats in a quartz tube with Ar flow. The porcelain boat with NaH_2PO_2 H_2O was put at the upstream side while $NiCo(OH)_2$/NF was placed at downstream side (the mass ratio of NaH_2PO_2 H_2O and $NiCo(OH)_2$ was 5:1). The quartz tube was heated up to 300 ℃, allowing the thermal decomposition of NaH_2PO_2 and the generation of PH_3 gas. The phosphidation was carried out at 300 ^oC for 120 min before cooling down to room temperature.

1.3 $Ni(OH)_2$/NiOOH electrode

The $Ni(OH)_2$ electrode comprised $Ni(OH)_2$, CoO and polytetrafluoroethylene (PTFE), carboxymethylcellulose (CMC) which bound to NF. The mass loading of $Ni(OH)_2$ on NF is ca. 330 mg cm^{-2} (~95 mhA cm^{-2}). The NiOOH electrode was prepared *in-situ* via electrochemical oxidation of $Ni(OH)_2$ electrode.

1.4 Rotating disk electrode (RDE) preparation

The glassy carbon RDE (Φ=5 mm, A: 0.19625 cm^2) was polished with 0.3 and 0.05 μm alumina powders, and rinsed sequentially with acetone, ethanol and DI water. The catalyst ink, which contained 5 mg of catalyst, 50 μL 5 wt% Nafion solution, 50 μL DI water and 900 μL ethanol, was sonicated for 2 h before use. The ink was drop-casted on the cleaned RDE (mass loading = 0.56 mg cm^{-2}) and then dried in air.

1.5 Gas diffusion electrode (GDE) and porous electrode (PE) preparation

GDE preparation. 20 mg catalyst and 200 μL 5 wt% Nafion solution was first mixed and ground with a pestle. After grinding for 10 min, 2 mL of isopropanol was added to the slurry for another 10 min grinding. The obtained suspension was transferred to a vial where additional 18 mL of isopropanol was added. After sonication for 2 h, a homogenous catalyst ink was obtained. The ink was sprayed onto the catalyst layer of the GDE with a mass loading of ca. 2 mg cm^{-2}.

PE preparation. PE was prepared by the same method that was used for making HER/OER bifunctional catalyst.

1.6 Electrolyzer assembly

As shown in supplementary Fig. 1, both compartments of electrolyzer comprised the PTFE end- and middle-plates, Ti-plate with flow-channel as the current collectors, GDEs, polypropylene-based separators, $Ni(OH)_2$/NiOOH electrode and PEs. 30 wt% KOH solution was used as the electrolyte. To avoid the mixture of O_2 and H_2 in the aqueous phase during current polarity swap, O_2-rich electrolyte and H_2-rich electrolyte were separately stored in two containers, which were circulated in the designated compartments by two peristaltic pumps (Longer, BT 100-3) at the flow rate of 15 ml min^{-1}. The CO_2 scrubber was a concentrated KOH (50 wt %) solution.

In the alternative design of the device, the major components were essentially identical with the design above. Yet, the function of abovementioned GDEs and PEs were combined in the new GDEs which were employed with four-functionally active catalysts (ORR, HOR, HER and OER). The schematic design and the assembly were detailed in supplementary Fig. 26.

2. Materials and electrode characterization

2.1 Materials characterizations

XRD patterns were obtained by using a D8 Advance X-ray diffractometer with Cu Kα radiation (λ=1.5418 Å) between 5° and 80°. The microstructure images were taken on an S-4800 field-emission scanning electron microscope coupled with an energy-disperse X-ray spectrometer (SEM, Hitachi, Japan). Transmission electron microscopy (TEM) and elemental mapping were collected by using a TEM (JEM-F200) equipped with an energy-disperse X-ray spectrometer (EDX). X-ray photoelectron spectroscopy (XPS) was carried out on a Thermo Fisher ESCALAB 250Xi instrument. Raman spectroscopy was recorded on an Invia/Reflrx Laser Micro-Raman spectroscope (Renishaw inVia, UK) with an equipped excitation laser beam wavelength of 532 nm. Gas purity was measured using an online gas chromatography (Shimadzu GC-2014C).

2.2 Electrocatalysts performance

The basic electrochemical measurements of electrocatalysts were performed in a standard three-electrode system using a CHI 760E electrochemical analyzer (CH Instrument Inc.). The ORR and HOR performance were performed in 0.1 M KOH using an RDE. Saturated calomel electrode (SCE) and a graphite rod was used as reference electrode and counter electrode, respectively. For ORR experiment, O_2 was bubbled into the electrolyte for 15 min prior to the test. Linear sweep voltammetry (LSV) was performed at a scan rate of 1 mV s^{-1} and at various rotating speeds from

400 to 2500 rpm. All the potentials were referenced to the reversible hydrogen electrode (RHE): $E_{RHE} = E_{SCE} + 0.242 + 0.059 \times pH$. Koutecky-Levich equation was used for calculating the electron transfer number (n):

$$\frac{1}{J} = \frac{1}{J_L} + \frac{1}{J_K} = \frac{1}{B\omega^{\frac{1}{2}}} + \frac{1}{J_K}$$

$$B = 0.62nFC_0 D_0^{2/3} v^{-1/6}$$

where J is the measured current density, J_K and J_L are the kinetic and diffusion-limiting
current densities, ω is the angular velocity, n is transferred electron number, F is the Faraday constant (96485.34 C mol^{-1}), C_0 is the bulk concentration of O_2 (1.2 × 10^{-6} mol cm^{-3}), D_0 is the diffusion coefficient of O_2 in 0.1 M KOH (1.9 × 10^{-5} cm^2 s^{-1}), and ν is the kinematic viscosity of the electrolyte (0.01 cm^2 s^{-1}).

For HOR experiment, H_2 was bubbled into the electrolyte for 15 min prior to the test. LSV was performed at a scan rate of 1mV s^{-1}. HER and OER test was tested in 1.0 M KOH. A piece of NCP/NF (1×1 cm) was directly used as the working electrode. The LSV was performed at 1 mV s^{-1}. All the electrochemical impedance spectra (EIS) were recorded at frequencies ranging from 10^5 Hz to 0.1 Hz. Post-iR compensation was applied in all electrochemical tests using 3-electrode setup.

2.3 Differential electrochemical mass spectrometry (DEMS)

Mass signals were recorded using PFEIFFER QAS100 DEMS with a turbo pump (HIPACE 80, PFEIFFER). The key parameters of electron impact ionization, electron energy and emission current are set as 70 eV and 2000 μA, respectively. The gold-plated porous PTFE film (commonly known as gold film, its thickness of 25 μm, porosity of 50%, pore size of 25 nm), which has permeability to volatile substances, was used to separate the electrolyte from the vacuum. Any volatiles generated from the electrode-electrolyte interface can be pumped into the mass spectrometer for analysis. The catalyst ink was coated on the gold film as the working electrode. 100 μL of catalyst ink was drop-casted onto the gold film in 10 additions of 10 μL. The working electrode area was 0.5 cm^2. The counter and reference electrodes are identical to those used in electrochemical measurements. Cyclic voltammetry (CV) was used to detect gaseous products. The voltage range of CV was 0.90~2.40 V vs. RHE with a scan speed of 10 mV s^{-1}.

3. Electrolyzer test

The electrochemical test of the electrolyzer (water electrolysis, Ni-H_2 battery and fuel cell) was conducted using a potentiostat (Autolab pgstat M204) coupled with a 10

A current booster. Pure humidified H_2 and O_2 gases (purity: 99.99%) were supplied by hydrogen and oxygen generators (Pulaixi, China), respectively. Compressed air was supplied from the cylinder. The flow rates of all gases were set at 200 mL min^{-1}. The test temperature was fixed at 50 ^{o}C. The volume and purity of the effluent gas was measured in the charging process of fuel cell. Other details are specified below.

3.1 Battery test

The power density plots of batteries (Ni-MH and Ni-H_2 battery) were calculated from the polarization curves at a scan rate of 5 mV s^{-1}. Galvanostatic charge-discharge measurements were performed by charging the batteries at different current densities (10 mA cm^{-2}, 20 mA cm^{-2} and 40 mA cm^{-2}). The cycle stability was carried out at a current density of 40 mA cm^{-2} with a cycling interval of 20 s. Externally-fed hydrogen was used during the discharging cycle.

The specific capacity was calculated according the equation below:

$$Specific\ capacity = \frac{current * service\ hours}{The\ mass\ of\ the\ active\ substance} \qquad (1)$$

where the active substance is $Ni(OH)_2$/ NiOOH.

The energy density was calculated according the equation below:

$$Energy\ density = \frac{capacity\ *\ average\ discharge\ voltage}{The\ mass\ of\ battery} \qquad (2)$$

where the mass of battery is consisted of the mass of GDEs, PEs, separators, electrolyte and consumed hydrogen.

The Coulomb efficiency was calculated according the equation below:

$$Coulomb\ efficiency = \frac{discharge\ capacity}{charge\ capacity} \qquad (3)$$

The round-trip energy efficiency was calculated according the equation below:

$$Round\ trip\ efficiency = \frac{discharge\ voltage}{charge\ voltage} * Coulomb\ efficiency \qquad (4)$$

The stored electrical energy was calculated according the equation below:

$$Stored\ electrical\ energy = charge\ voltage * current * charging\ time \qquad (5)$$

The released electrical energy was calculated according the equation below:

$$Released\ electrical\ energy = \ discharge\ voltage * curren * discharging\ time \qquad (6)$$

The depth of discharge (DoD) was calculated according the equation below:

$$Depth\ of\ discharge = \frac{charged/discharged\ capacity}{theoretical\ \ capacity} \qquad (7)$$

3.2 Fuel cell and electrolyzer test

In the Fuel cell test, the polarization curve and the power density plot were obtained at a scan rate of 5 mV s^{-1} (cut-off voltage: 0.3). The current polarity was reversed once the capacity of the NiOOH/$Ni(OH)_2$ auxiliary electrode reached 50 mAh cm^{-2} (150 mAh g^{-1}, ca. 52.6% DoD). The flow of O_2-rich and H_2-rich electrolyte was also swapped in the meantime.

The energy efficiency of a fuel cel, ε_{FC}, was calculated according the equation below:

$$\varepsilon_{discharge} = \varepsilon_E * \varepsilon_F * \varepsilon_{cell}^{rev} \quad (8)$$

ε_F is the fuel utilization efficiency (assuming 100% in our calculation); ε_E is the voltage efficiency ($\frac{E_{cell}}{E_{rev}}$); $E_{battery}$ is the battery potential at operation; E_{rev} is the reversible battery voltage at standard condition (1.23 V); ε_{cell}^{rev} is the thermodynamic efficiency, $\varepsilon_{cell}^{rev} = \frac{\Delta rG_{rev}}{\Delta rH_{rev}} = \frac{237.17\ KJ\ mol^{-1}}{285.83\ KJ\ mol^{-1}} = 83.0\%$. The energy consumed by the auxiliary equipment such as the pump and the degassing process was not considered.

The released electrical energy was calculated according the equation below:

$$\text{released electrical energy} = \text{operation voltage} * \text{current} * \text{operation time}$$

In the water electrolysis test, the polarization curve was obtained at 1 mV s^{-1}. The current polarity was reversed once the capacity of the NiOOH/$Ni(OH)_2$ auxiliary electrode reached 50 mAh cm^{-2} (ca.52.6 % DoD).

The energy efficiency of the electrolyzer, ε_{EC}, was calculated according the equation below:

$$\varepsilon_{charge} = \frac{1}{1 + \frac{nF(U_{battery} - U_{rev})}{\Delta rH_{rev}}} \approx \frac{U_{cell}}{U_{rev}} * \varepsilon_F \quad (9)$$

where n is the stoichiometric charge number; U_{rev} is the reversible cell voltage (1.23 V); F is the Faraday constant (F = 96485.34 C mol^{-1}); U_{cell} is the cell voltage at operation; ε_F is the Faradaic efficiency; the energy consumed by the auxiliary equipment such as the pump and the degassing process was not considered.

The Faradaic efficiency was calculated according to the following equation:

$$\varepsilon_F = \frac{n(gas)_{measured}}{n(gas)_{theoretical}} = \frac{P \cdot V(gas)_{measured}/RT}{Q/nF} \quad (10)$$

Wherein P is the pressure (1 atm), V is the volume of the effluent gas, R is the gas constant (8.314 J mol^{-1} K^{-1}), T is the temperature (298 K), $Q = \int I dt$ is the charge, n is the stoichiometric charge number (2 electrons per H_2 molecule and 4 electrons per O_2 molecule).

The stored electrical energy was calculated according the equation below:

$$\text{Stored electrical energy} = \Delta c H_m^{\theta}(H_{2,}\ g, 298\ K) * n(H_2)_{measured} \quad (11)$$

where $\Delta c H_m^{\theta}(H_2,\ g,\ 298\ K)$is the absolute value of the standard molar enthalpy of hydrogen combustion (285.83 kJ mol^{-1}); $n(H_2)_{measured}$ is measured moles of hydrogen.

4. Supplementary Figures

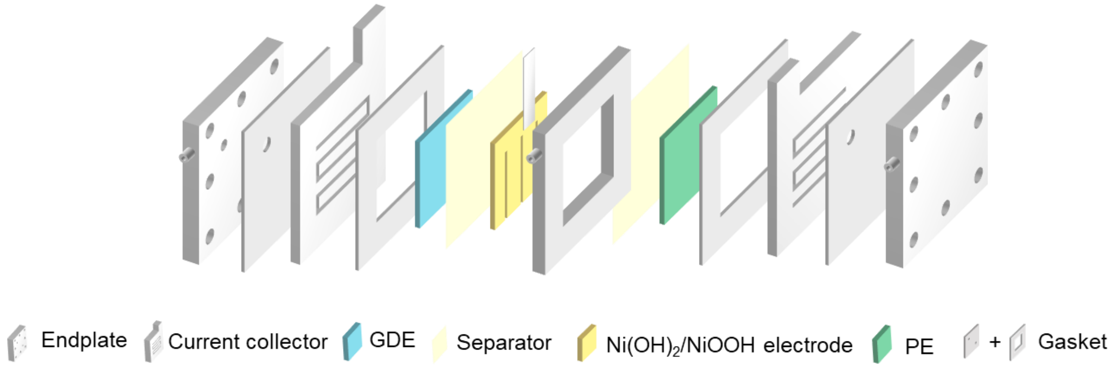

Supplementary Figure 1. The schematic assembly of the electrolyzer.

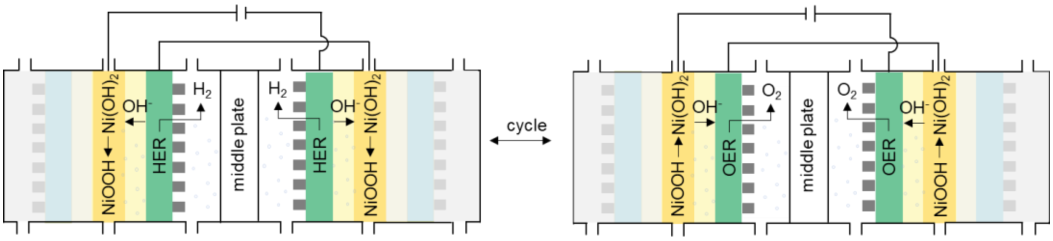

Supplementary Figure 2. (a) The working principles in low current density water electrolysis mode.

In low-load splitting water mode, both compartments were connected in series. During hydrogen evolution step, the PEs of the two compartments catalyze HER to produce H_2 and OH^-. The generated OH^- ions pass through the separator and react with Ni $(OH)_2$ to form NiOOH. When the redox couple conversion reached the setpoint, the potential bias between the two compartments was swapped to enable the reverse reaction of the auxiliary electrode and the working electrode. Subsequently, the both compartments becomes the oxygen compartment. During oxygen evolution step, OER occurs on the PE in the O_2 compartment where NiOOH was converted into $Ni(OH)_2$.

Hydrogen evolution step

Anode: $$Ni(OH)_2 + OH^- \longrightarrow NiOOH + H_2O + e^- \quad (12)$$

Cathode: $$H_2O + e^- \xrightarrow{PE} \frac{1}{2}H_2 + OH^- \quad (13)$$

Overall: $$Ni(OH)_2 \longrightarrow NiOOH + \frac{1}{2}H_2 \quad (14)$$

Oxygen evolution step

Anode: $$OH^- \xrightarrow{PE} \frac{1}{4}O_2 + \frac{1}{2}H_2O + e^- \quad (15)$$

Cathode: $$NiOOH + H_2O + e^- \rightarrow Ni(OH)_2 + OH^- \quad (16)$$

Overall: $$NiOOH + \frac{1}{2}H_2O \longrightarrow Ni(OH)_2 + \frac{1}{4}O_2 \quad (17)$$

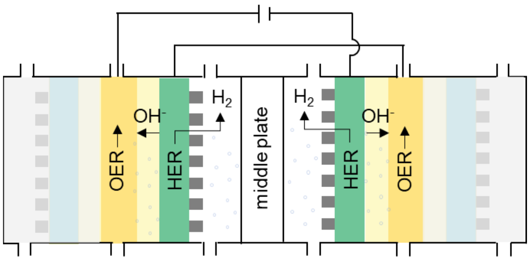

Supplementary Figure 3. The working principles in low-load splitting water mode**.**

In high-load splitting water mode, both compartments were also connected in series. The PEs of the two compartments catalyze HER to produce H_2 and OH^-. At the same time, NiOOH generated on the auxiliary electrode can catalyze OER on the anode.

Anode: $$OH^- \xrightarrow{AE} \frac{1}{4}O_2 + \frac{1}{2}H_2O + e^- \quad (15)$$

Cathode: $$H_2O + e^- \xrightarrow{PE} \frac{1}{2}H_2 + OH^- \quad (13)$$

Overall: $$\frac{1}{2}H_2O \longrightarrow \frac{1}{4}O_2 + \frac{1}{2}H_2 \quad (18)$$

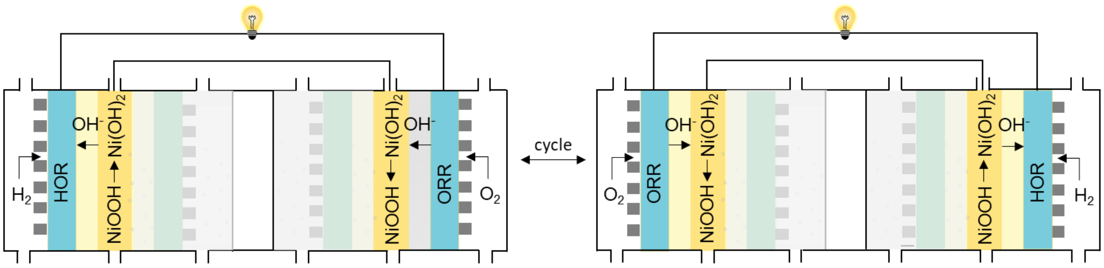

Supplementary Figure 4. The working principles in fuel cell mode.

During fuel cell mode for the long-duration storage, the hydrogen compartment is the Ni-H_2 battery as detailed above; in the oxygen compartment, ORR takes place on the anode which offers OH^- ions for the $Ni(OH)_2$-NiOOH conversion. When the redox couple conversion reached the setpoint, the potential bias between the two compartments was swapped to enable the reverse reaction of the auxiliary electrode and the working electrode. Subsequently, the left compartment becomes the oxygen compartment while the right one becomes the hydrogen compartment.

H_2 compartment:

Cathode: $$\frac{1}{2}H_2 + OH^- \rightarrow H_2O + e^- \quad (19)$$

Anode: $$NiOOH + H_2O + e^- \rightarrow Ni(OH)_2 + OH^- \quad (16)$$

O_2 compartment:

Cathode: $$\frac{1}{4}O_2 + \frac{1}{2}H_2O + e^- \rightarrow OH^- \quad (20)$$

Anode: $$Ni(OH)_2 + OH^- \rightarrow NiOOH + H_2O + e^- \quad (12)$$

Overall: $$\frac{1}{2}H_2 + \frac{1}{4}O_2 \rightarrow \frac{1}{2}H_2O \quad (21)$$

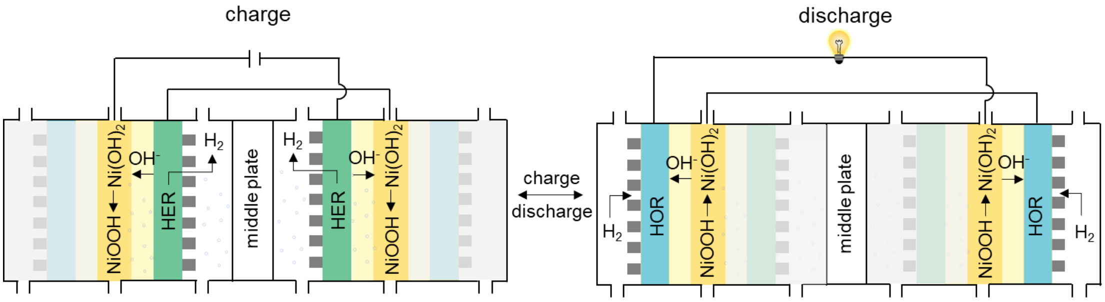

Supplementary Figure 5. The working principles in Ni-H_2 battery mode.

In Ni-H_2 battery mode for the short-duration storage, both compartments become the classical Ni-H_2 batteries connected in series. During charging, the PEs of the two batteries catalyze HER to produce H_2 and OH^-. The generated OH^- ions pass through the separator and react with Ni $(OH)_2$ to form NiOOH. During discharging, NiOOH is reduced to Ni $(OH)_2$ and OH^-, while the H_2 in the storage tank goes to GDE where the HOR occurs.

Anode: $$H_2O + e^- \underset{\text{discharge}}{\overset{\text{charge}}{\rightleftharpoons}} \frac{1}{2}H_2 + OH^- \quad (22)$$

Cathode: $$Ni(OH)_2 + OH^- \underset{\text{discharge}}{\overset{\text{charge}}{\rightleftharpoons}} NiOOH + H_2O + e^- \quad (23)$$

Overall: $$Ni(OH)_2 \underset{\text{discharge}}{\overset{\text{charge}}{\rightleftharpoons}} NiOOH + \frac{1}{2}H_2 \quad (24)$$

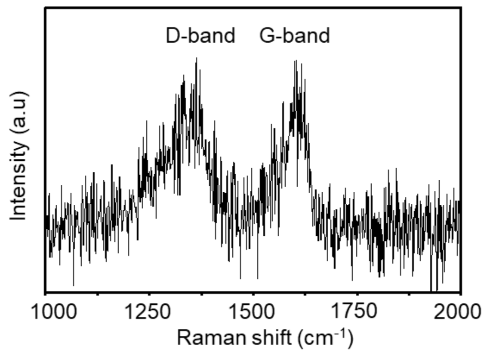

Supplementary Figure 6. The Raman spectra of Pt/NC/ NCC.

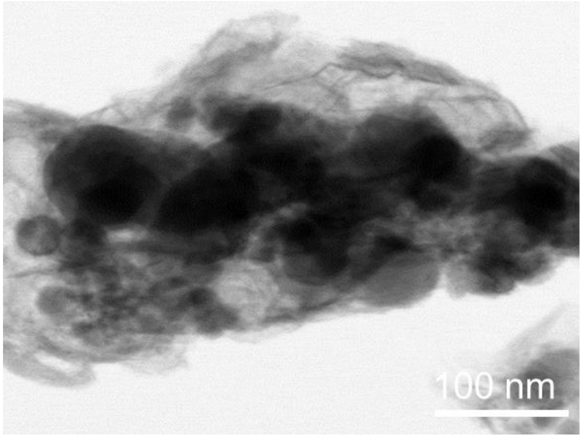

Supplementary Figure 7. The TEM images of Pt/NC/NCC.

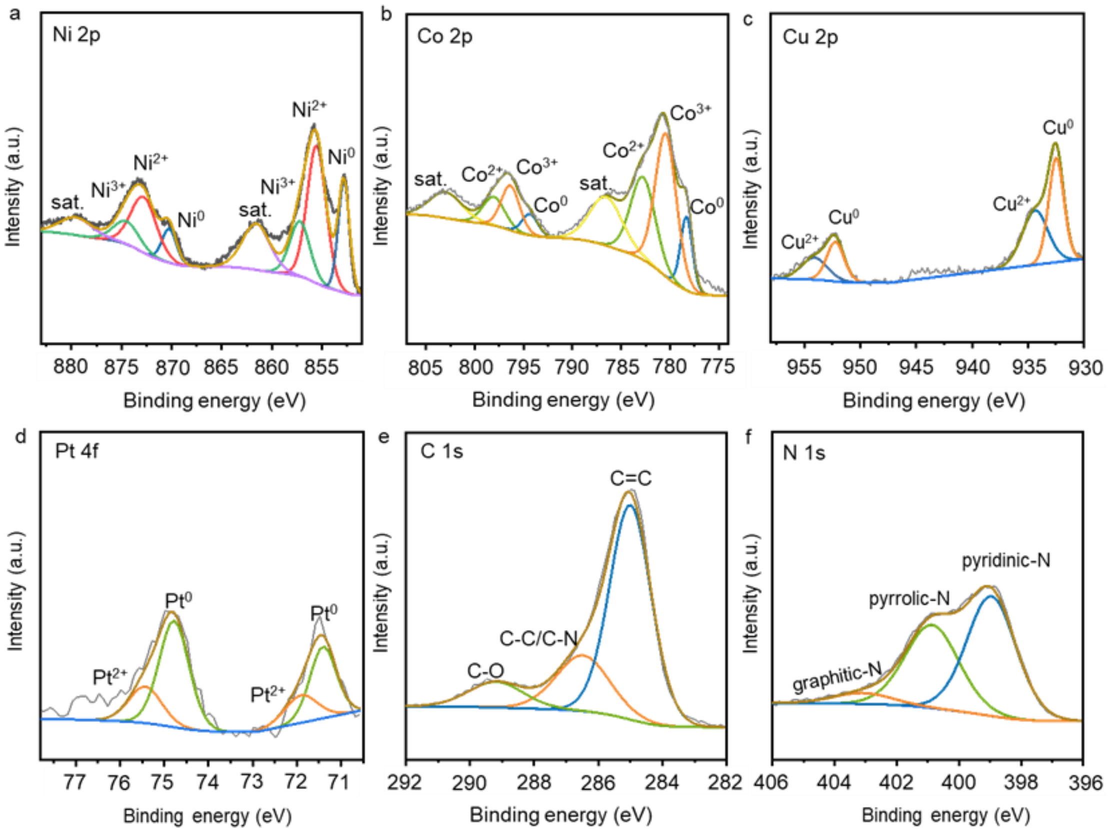

Supplementary Figure 8. XPS spectra for (a) Ni 2p, (b) Co 2p, (c) Cu 2p, (d) Pt, (c) C 1s and N 1s.

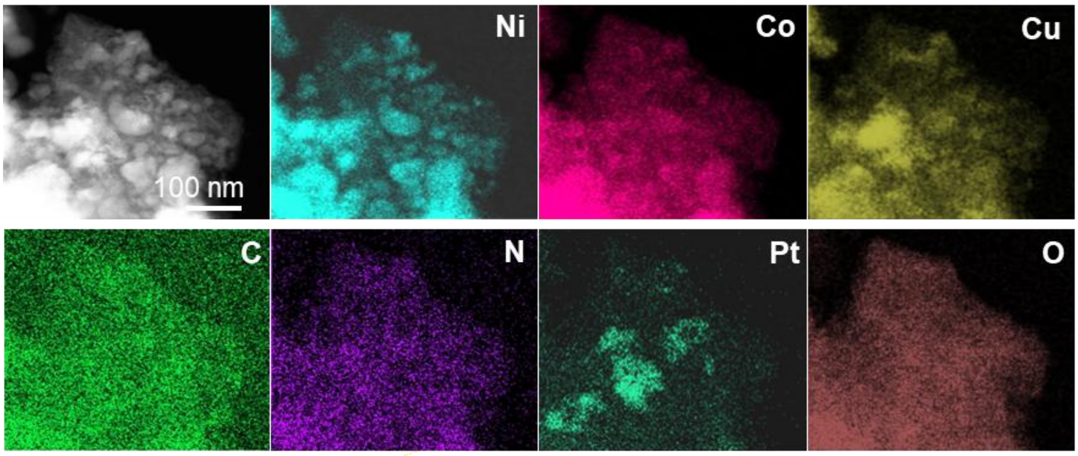

Supplementary Figure 9. EDX mapping of Pt/NC/NCC.

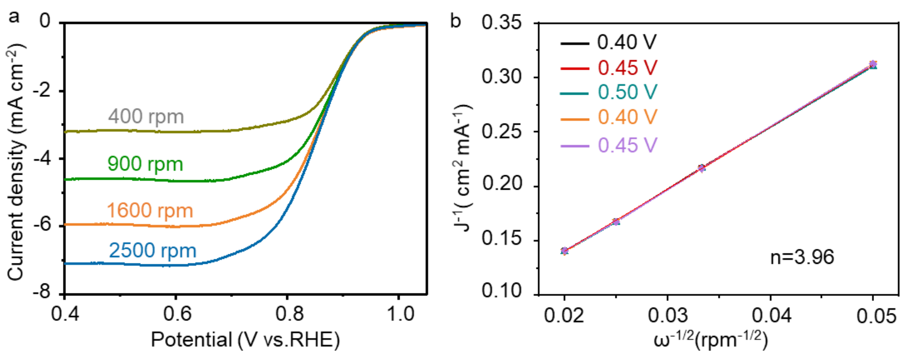

Supplementary Figure 10. (a) Polarization curves of Pt/NC/NCC at different rotation rates (400, 900, 1600 and 2500 rpm) in O_2-saturated 0.1 M KOH, (b) Koutecky ́– Levich plot based on RDE data.

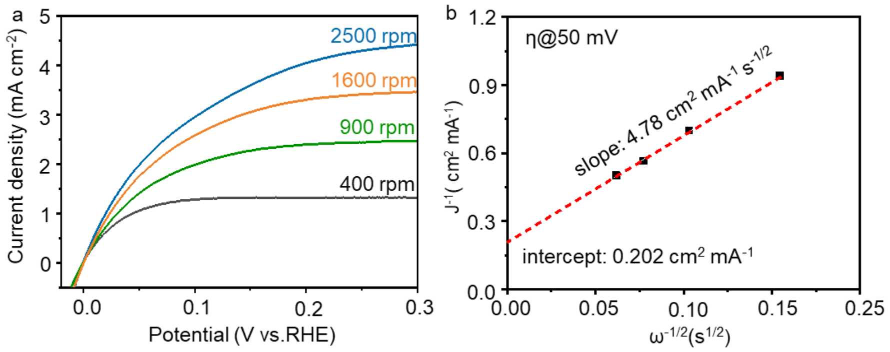

Supplementary Figure 11. (a) Polarization curves of Pt/NC/NCC at different rotation rates (400, 900, 1600 and 2500 rpm) in H_2-saturated 0.1 M KOH, (b) the fitting result of electrochemical data to Koutecky-Levich equation at an overpotential of 50 mV.

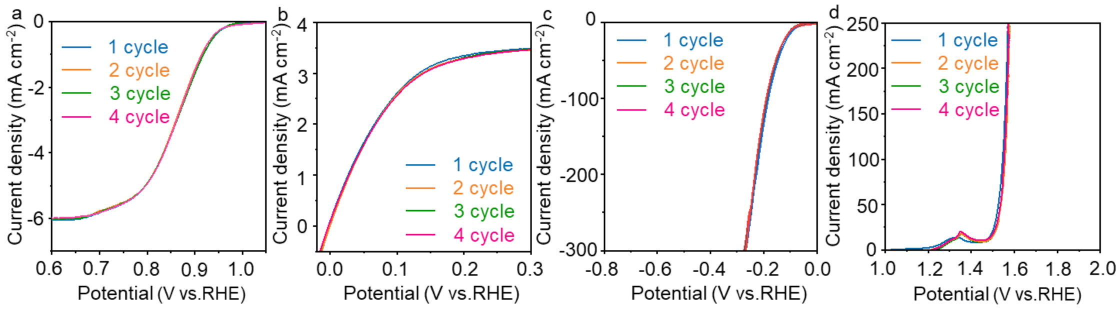

Supplementary Figure 12. (a, b) the LSV curves for Pt/NC/NCC after ORR-HOR cycles, (c, d) LSV curves for PE (NCP/NF) after HER-OER.

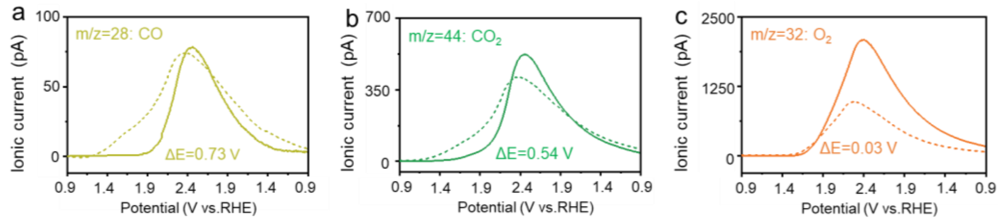

Supplementary Figure 13. (a, b, c) the DEMS signals using Pt/C (dotted line) and Pt/NC NCC (solid line).

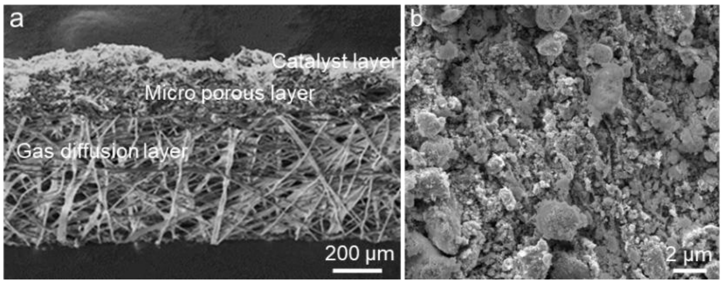

Supplementary Figure 14. (a, b) scanning electron micrographs of the GDE.

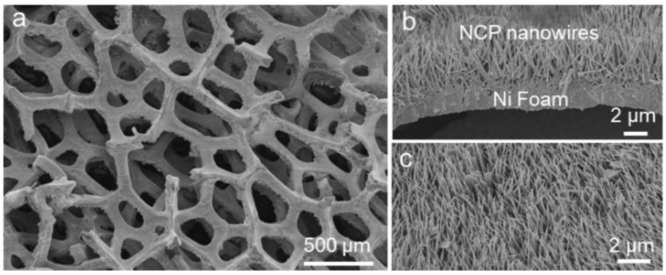

Supplementary Figure 15. (a, b, c) scanning electron micrographs of the porous electrode.

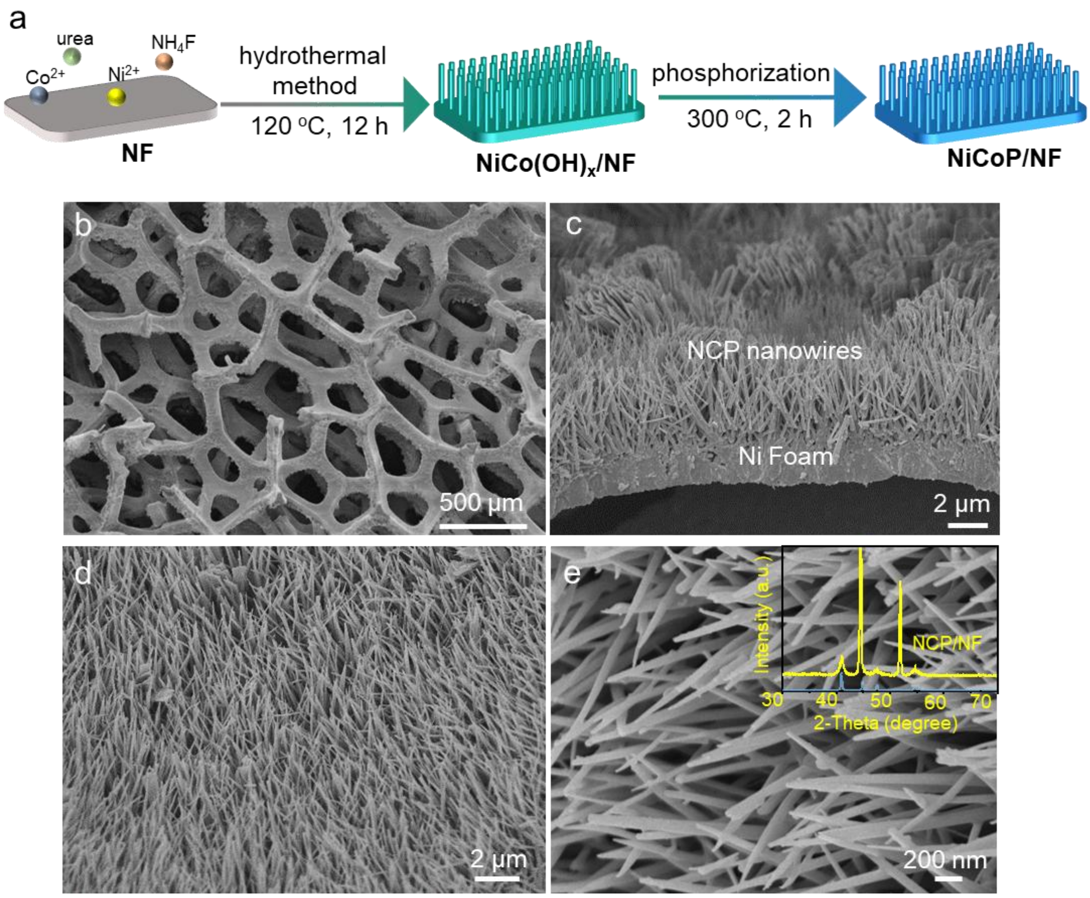

Supplementary Figure 16. (a) the synthetic procedure of the porous electrode, **(**b) SEM images, (c, d) cross-sectional and surface images, (e) enlarged images of d (inset, XRD pattern of the porous electrode).

With feeding oxygen:

Anode: $$Ni(OH)_2 + OH^- \rightarrow NiOOH + H_2O + e^- \qquad E^0=+0.49 \qquad (26)$$

Cathode: $$\frac{1}{4}O_2 + \frac{1}{2}H_2O + e^- \rightarrow OH^- \qquad E^0=+0.41 \qquad (27)$$

Overall: $$Ni(OH)_2 + \frac{1}{4}O_2 \rightarrow NiOOH + \frac{1}{2}H_2O \qquad E^0=-0.09 \qquad (28)$$

Without feeding oxygen:

Anode: $$Ni(OH)_2 + OH^- \rightarrow NiOOH + H_2O + e^- \qquad E^0=+0.49 \qquad (29)$$

Cathode: $$H_2O + e^- \rightarrow \frac{1}{2}H_2 + OH^- \qquad E^0=-0.83 \qquad (30)$$

Overall: $$Ni(OH)_2 + e^- \rightarrow NiOOH + \frac{1}{2}H_2 \qquad E^0=+1.32 \qquad (31)$$

Supplementary Figure 17. the detailed reactions of oxygen compartment operation under different conditions.

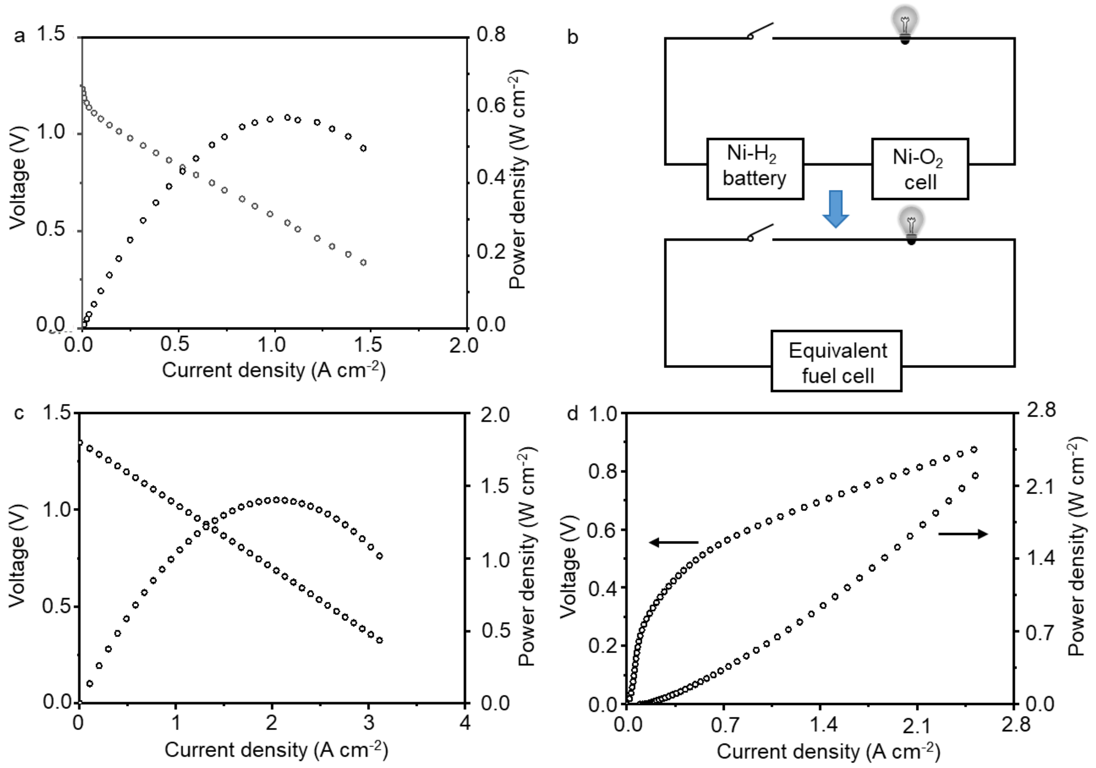

Supplementary Figure 18 (a) The calculated polarization and power density curves of the SMAWE working as fuel cell, (b) the equivalent circuit diagram of the Ni-H_2 battery and Ni-O_2 cell connected in series, (c, d) the polarization and power density curves of Ni-H_2 battery and Ni-O_2 cell used for the calculated fuel cell performance in (a).

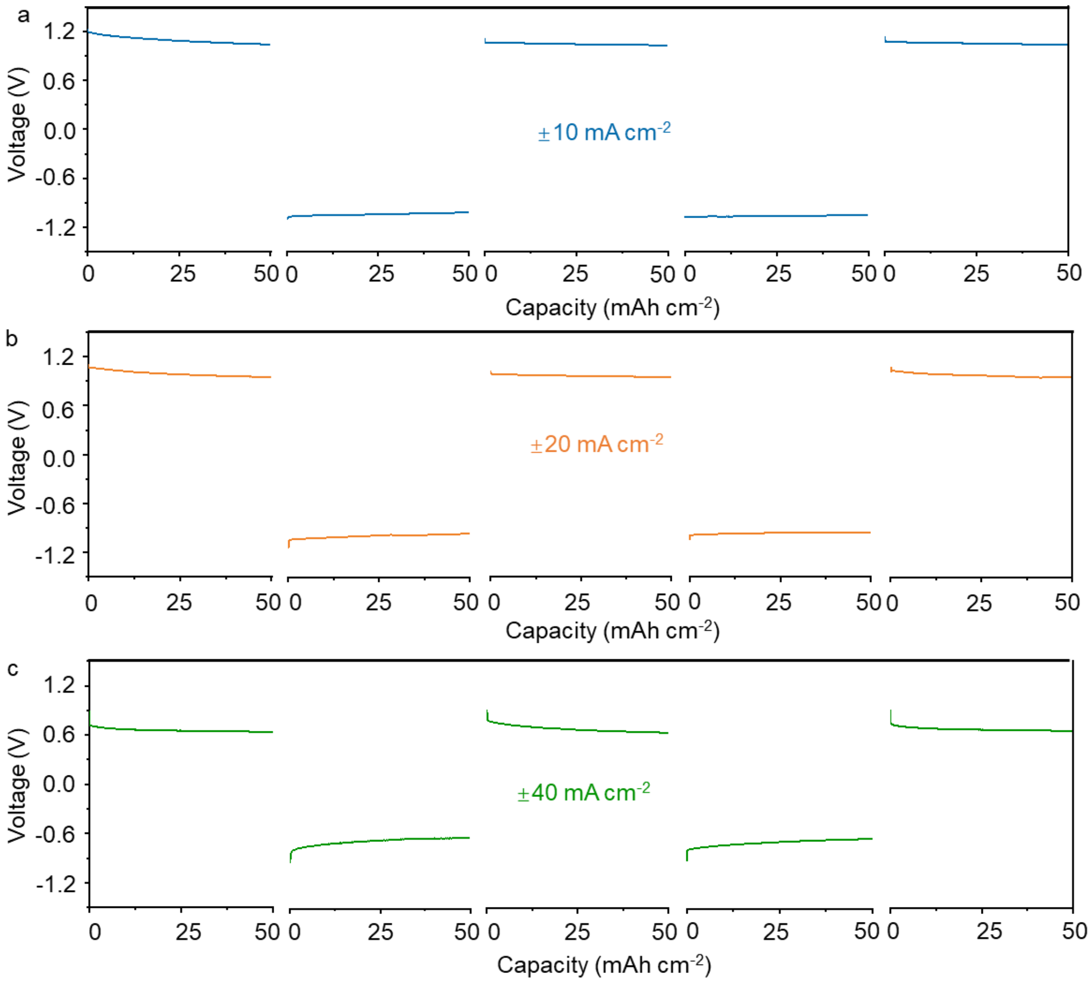

Supplementary Figure 19. Cyclic operation of the SMAWE in fuel cell mode at: (a) ±10 mA cm^{-2}, (b) ±20 mA cm^{-2}, (c) ±40 mA cm^{-2}.

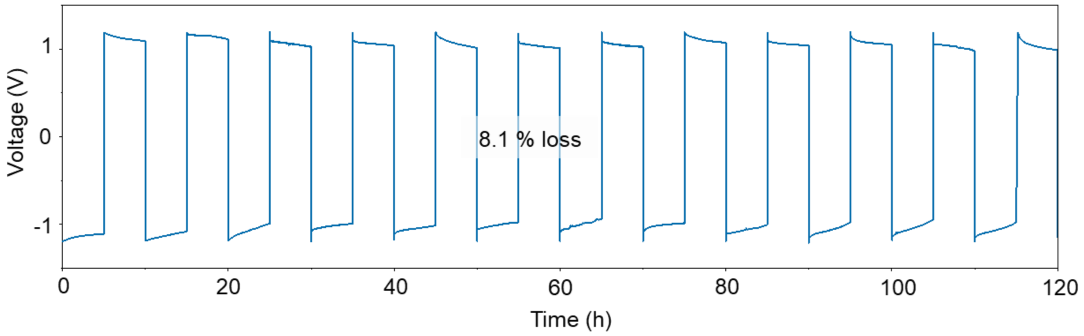

Supplementary Figure 20. Cyclic stability at ±10 mA cm^{-2} in fuel cell mode.

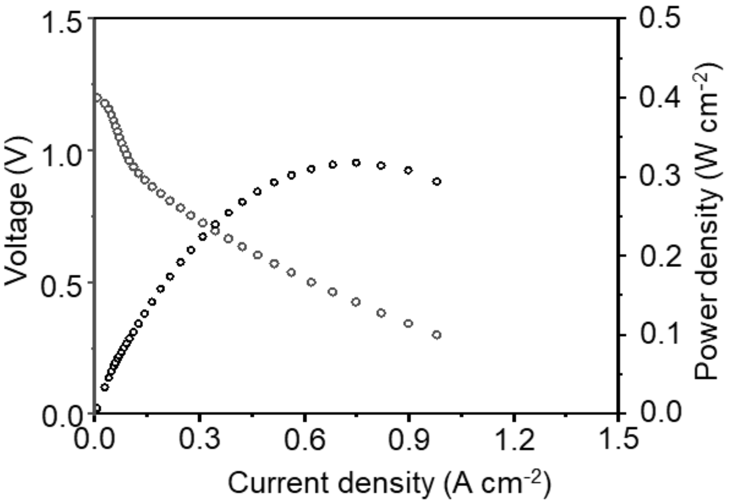

Supplementary Figure 21. (a) the polarization and power density curve of fuel cell mode using pure H_2 and CO_2-free air.

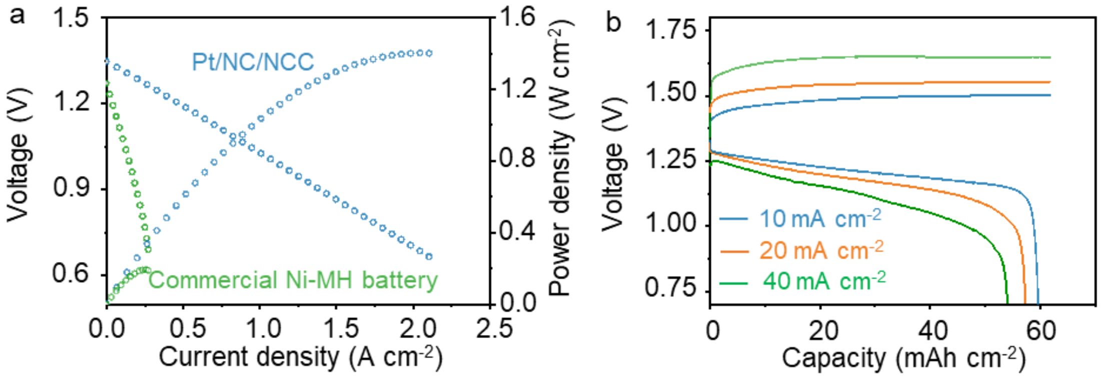

Supplementary Figure 22. polarization and power density curve in Ni-H_2 battery mode.

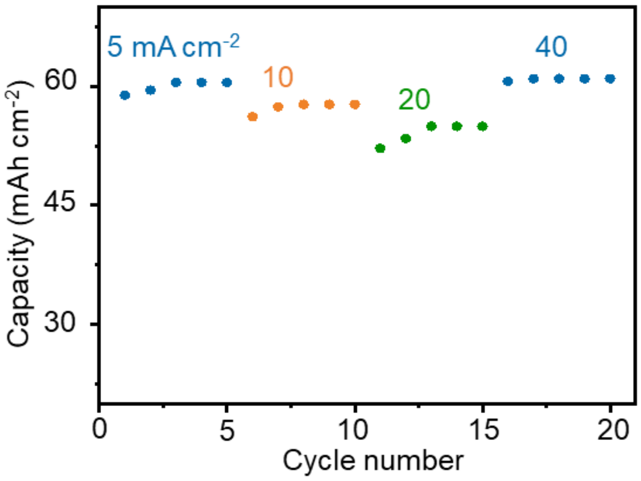

Supplementary Figure 23. Rate capability of the Ni-H_2 battery.

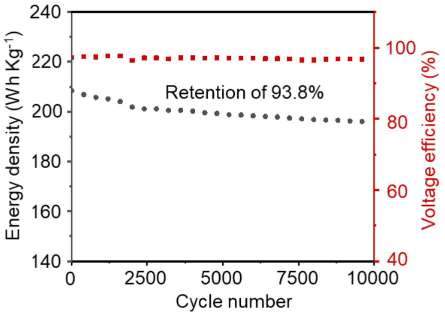

Supplementary Figure 24. Cycle charge/discharge in Ni-H_2 battery at a constant current of 40 mA cm^{-2} (charge/discharge capacity: 0.444 mAh cm^{-2}).

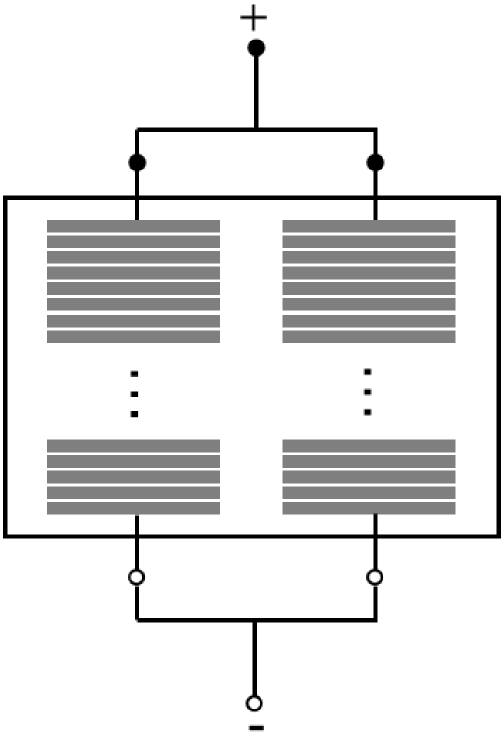

Supplementary Figure 25. The configuration of the membrane-free alkaline water electrolyzer stack which is comprised of two sub-stacks. When one sub-stack is working as the oxygen stack, the other one operates as the hydrogen stack in fuel cell mode.

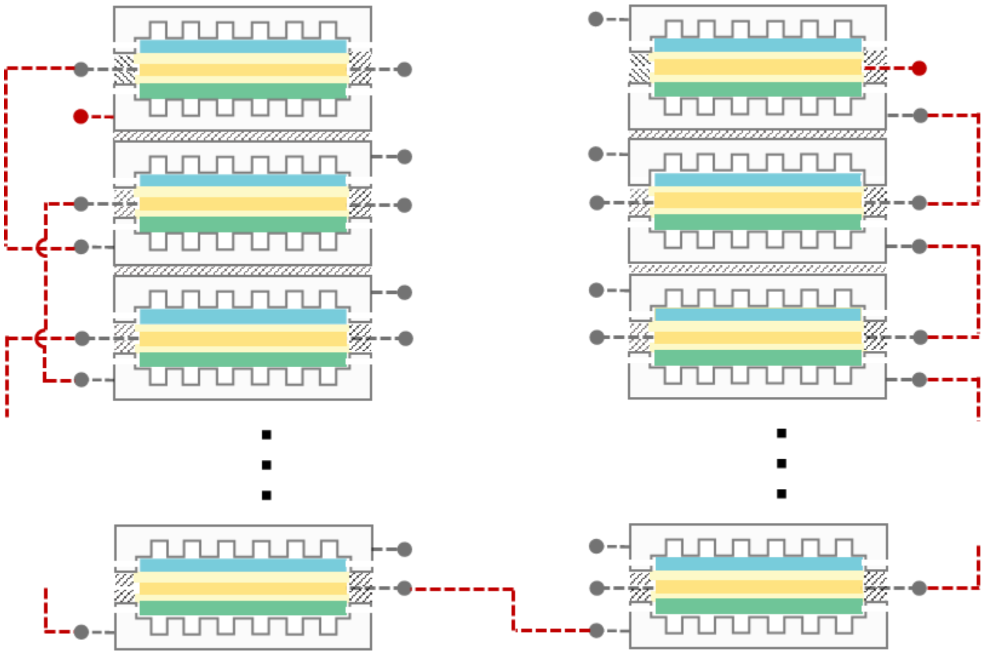

Supplementary Figure 26. The membrane-free alkaline water electrolyzer stack configuration in water electrolysis mode with electron flows.

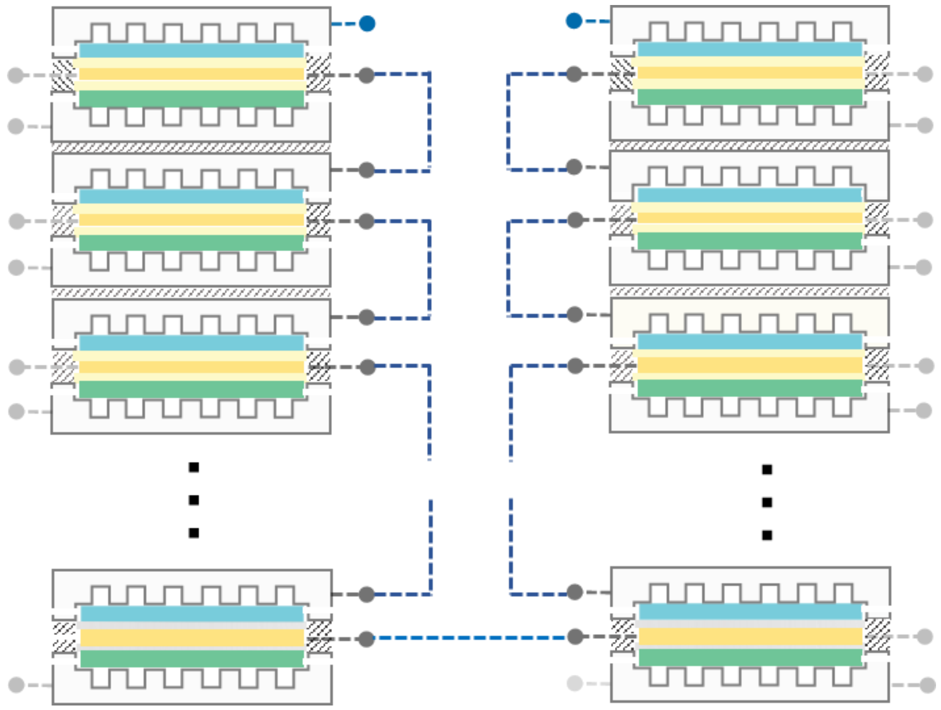

Supplementary Figure 27. The membrane-free alkaline water electrolyzer stack configuration in fuel cell mode with electron flows.

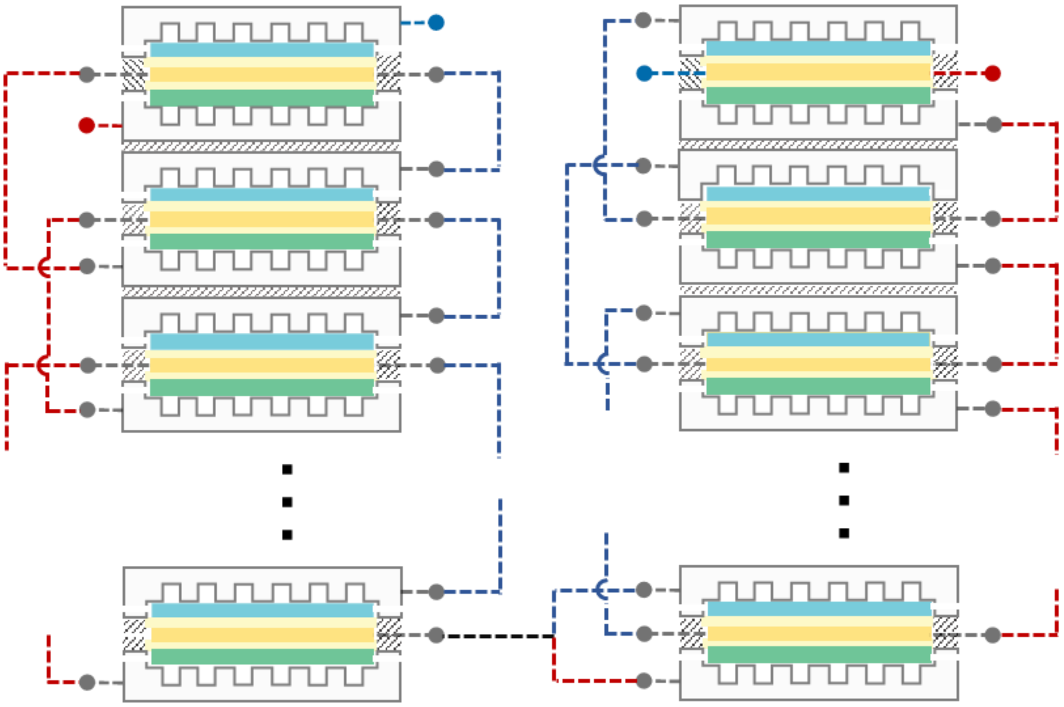

Supplementary Figure 28. The membrane-free alkaline water electrolyzer stack configuration in Ni-H_2 battery mode with electron flows.

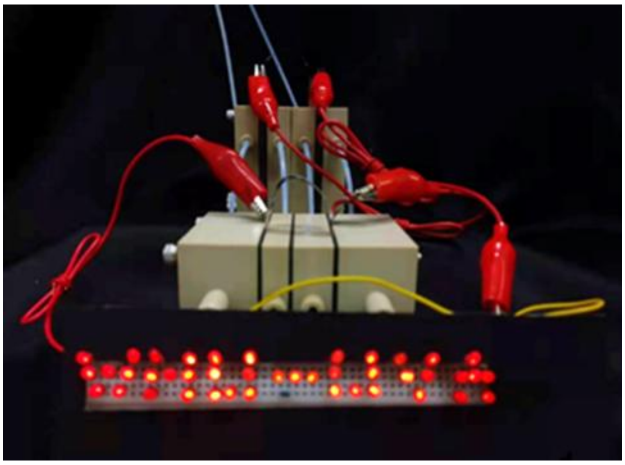

Supplementary Figure 29. Photograph of red LEDs powered an electrolyzer.

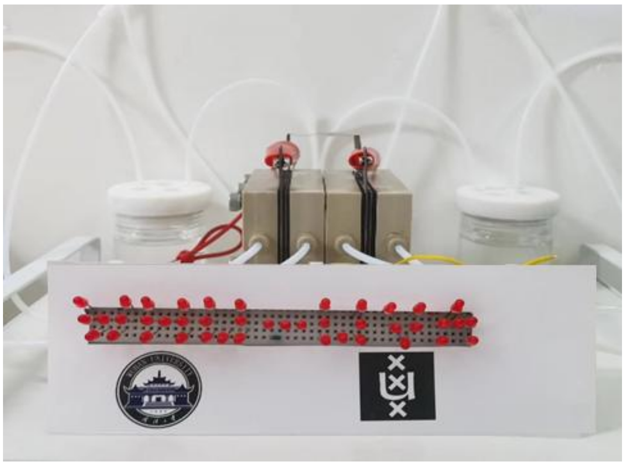

Supplementary Figure 34. Photograph of the LEDs when the electrolyzer is off.

5. Cost evaluation

5.1 Electrolyzer cost

The total capital cost of a alkaline water electrolyzer is evaluated on the basis of both the storage cost (US $ kWh-1) and the power conversion cost (US $ kW^{-1}). The power conversion cost (C_{PCC}) was calculated as follows:

$$C_{PCC} = \frac{\sum_n p_n A_n}{P * 22.3\%}$$

where P is the power of the electrolyzer. We selected the power at 250 mA cm^{-2} (0.275 W cm^{-2}) as a reference; p is unit price of the electrode assembly (GDE, $Ni(OH)_2$/NF, PE and separator) in Supplementary table 2. A is the area of each electrode assembly component. The electrode assembly cost accounts for ~22.3% of system cost. This estimation was based on the data of the typical alkaline water electrolyzer which share the similar structure of the electrode assembly.[S1] Thus, the calculated C_{PCC} was ~160 US $ kW^{-1}).

5.2 Energy storage cost comparison

We compared the total capital cost of the electrolyzer with three major electrochemical energy storage technologies (i.e., AEMEC-AEMFC, PEMEC-PEMFC and SOEC-SOFC). The total cost is assessed based on hydrogen production cost (HPC), hydrogen storage cost (HSC) and hydrogen utilization cost (HUC).

$$\text{Total capital cost} = \text{HPC} + \text{HSC} + \text{HUC}$$

We considered a model system with the rated power of 100 MW, the storage duration is 100 h. Note that the total cost for AEMEC-AEMFC as calculated differently as detailed below.

5.2.1 AEMEC-AEMFC

The HPC, HSC and HUC we used in the calculation are 126.9 $ kW^{-1} (AEMEC), 81.6 $ kW^{-1} (AEMFC) and 20 $ kWh^{-1} (H_2 storage), respectively. The exemplified calculation was shown below:

For 50 h duration:

$$\text{HPC} = \$\ 1.27 \text{ million}$$

$$\text{HSC} = 20\ \$\ \text{kWh}^{-1} * 50 = \$\ 10 \text{ million}$$

$$\text{HUC} = \$\ 0.82 \text{ million}$$

$$\text{Total capital cost} = \$12.09 \text{ million}$$

5.2.2 PEMEC-PEMFC

The HPC and HUC we used in the calculation are 253 $kW^{-1}$and 249.72 $ kW, respectively. The exemplified calculation was shown below:

For 50 h duration:

$$\text{HPC} = 209\ \$\ \text{kWh}^{-1} * 5 = \$\ 2.53\ \text{million}$$

$$\text{HSC} = 20\ \$\ \text{kWh}^{-1} * 50 = \$\ 10\ \text{million}$$

$$\text{HUC} = \$\ 2.50\ \text{million}$$

$$\text{Total capital cost } = 10.45 + 4.79 = \$\ 15.03\ \text{million}$$

5.2.3 Reversible SOFC

The HPC and HUC we used in the calculation are 0 $kW^{-1}$and 153.59 $ kW, respectively. The exemplified calculation was shown below:

For 50 h duration:

$$\text{HPC} = 0\ \text{million}$$

$$\text{HSC} = 20\ \$\ \text{kWh}^{-1} * 50 = \$\ 10\ \text{million}$$

$$\text{HUC} = \$\ 1.54\ \text{million}$$

$$\text{Total capital cost } = 10.45 + 4.79 = \$\ 11.54\ \text{million}$$

5.2.4 Reversible SOFC

The HPC and HUC we used in the calculation are 0 kW^{-1} and 160 $ kW, respectively. The exemplified calculation was shown below:

For 50 h duration:

$$\text{HPC} = 0\ \text{million}$$

$$\text{HSC} = 20\ \$\ \text{kWh}^{-1} * 50 = \$\ 10\ \text{million}$$

$$\text{HUC} = \$\ 1.6\ \text{million}$$

$$\text{Total capital cost } = 10.45 + 4.79 = \$\ 11.6\ \text{million}$$

Supplementary Table 1. **The cost of chemicals and materials.**

**Chemical/Materials**	**Price**	**Unit**	**Source**
Pt	33850.4	$ kg^{-1}	https://www.dailymetalprice.com/
Ni	27.9	$ kg^{-1}	
Co	75.0	$ kg^{-1}	
Mo	66.0	$ kg^{-1}	

Cu	9.5	$ kg^{-1}	
Ir	167886		
Carbon black	0.04	$ kg^{-1}	https://www.chemanalyst.com/
Separator	2	$ m^{-2}	S2
Ni Foam (NF)	6	$ m^{-2}	S3
Gas diffusion layer (GDL)	3.82	$ m^{-2}	S4
Proton exchange membrane (PEM)	350	$ m^{-2}	https://www.fuelcellstore.com/product/product&product_id=2044
Anion exchange membrane (AEM)	220	$ m^{-2}	
TiO_2	3.49	$ kg^{-1}	https://businessanalytiq.com/procurementanalytics/index/titanium-dioxide-tio2-china-price-index/
NiO	14	$ kg^{-1}	https://www.chemanalyst.com/
ZrO_2	45.8	$ kg^{-1}	
$La_{0.8}Sr_{0.2}MnO_{3-x}$	7000	$ kg^{-1}	

Supplementary Table 2. The material cost of single compartment.

**Chemical/Materials**	**Mass loading**	**Component/ Price**
Gas diffusion electrode (GDE, Pt-NCC@NC)	2 mg cm^{-2}	GDE price: 0.00182 $ cm^{-2}
$Ni(OH)_2$/NF	333 mg cm^{-2}	$Ni(OH)_2$/NF price: 0.00653 $ cm^{-2}
PE (NCP/NF)	5.3 mg cm^{-2}	PE price: 0.000838 $ cm^{-2}
Separator	n.g.	Separator price: 0.0002 $ cm^{-2}
Pt/C/carbon paper (CP) [S5]	0.4 mg_{Pt} cm^{-2}	Pt/C/CP price: 0.0139 $ cm^{-2}
IrO_2/CP [S6]	0.1 mg_{Ir} cm^{-2}	IrO_2/CP price: 0.0172 $ m^{-2}
PEM	n.g.	PEM price: 0.0594 $ cm^{-2}
Pt/C	0.1/0.2 mg_{Pt} cm^{-2}	$Pt_{0.1}$/C price: 0.0034/0.0068 $ cm^{-2}
Fe_2P-$Ni_{12}P_5$/NF[S7]	3 mg cm^{-2}	Fe_2P-$Ni_{12}P_5$/NF price:0.0349 $ cm^{-2}
Co_2P-$Ni_{12}P_5$/NF	3 mg cm^{-2}	Fe_2P-$Ni_{12}P_5$/NF price:0.0349 $ cm^{-2}
Ni+YSZ [S8]	n.g	Ni+YSZ price: 0.0815 $ cm^{-2}
LSM	n.g	LSM price: 0.0538

Note: 1. The price of chemicals come from Supplementary Table 1.
2. n.g. indicates not given.

Supplementary Table 3. The power conversion cost of a single electrolyzer with unit area.

**Parameters**	**GDE**	**$Ni(OH)_2$/NF**	**PE**	**Separator**
**Price ($ cm^{-2})**	0.00182	0.00653	0.000838	0.0002
**Area (cm^2)**	1*1			1.2*1.2
**MEA cost ($)**	0.009796 $			
**P (W)**	0.275			
**Total MEA cost ($ kW^{-1})**	35.6			
**Power conversion cost ($ kW^{-1})**	**160**			

Note: The price of each component comes from Supplementary Table 2.

6. Supplementary References

S1. Lagadec M. F., Grimaud A. Water electrolysers with closed and open electrochemical systems. Nat. Mater. 2020; 19: 1140-1150.

S2. Ho Nguyen D., et al. Design of portable hydrogen tank using adsorption material as storage media: An alternative to Type IV compressed tank. Appl. Energy. 2022; 310: 118552-118568.

S3. Kear G., et al. Development of the all-vanadium redox flow battery for energy storage: a review of technological, financial and policy aspects. Adv. Mater. Interfaces. 2012; 36: 1105-1120 (2012).

S4. Aravindan V., et al. Insertion-type electrodes for nonaqueous Li-ion capacitors. Chem. Rev. 2014; 114: 11619-11635.

S5. Yeo K., et al. A highly active and stable 3D dandelion spore-structured self-supporting Ir-based electrocatalyst for proton exchange membrane water electrolysis fabricated using structural reconstruction. Energy Environ. Sci.2022; 15: 3449-3462.

S6. Cheng Q., High-loaded sub-6 nm Pt_1Co_1 intermetallic compounds with highly efficient performance expression in PEMFCs. Energy Environ. Sci. 2022; 15: 278-287.

S7. Guo L., et al. Self-supported bimetallic phosphide heterojunction-integrated electrode promoting high-performance alkaline anion-exchange membrane water electrolysis. ACS Sustainable Chem. Eng.2022; 10: 9956−9968.

S8. Wang Z., et al. Preparation and characterization of silver-modified $La_{0.8}Sr_{0.2}MnO_3$ cathode powders for solid oxide fuel cells by chemical reduction method. Int. J. Hydrogen Energy.2013; 38; 1074-1081.
